# Preliminary evaluation of a DDA cationic liposome-based pulmonary mucosal immunization platform carrying a SARS-CoV-2 spike-derived branched peptide

**DOI:** 10.3389/fimmu.2026.1824741

**Published:** 2026-05-29

**Authors:** Zhifa Xia, Zhenyong Yang, Zhenwei Shi, Songtao Huang, Fenghua Xu

**Affiliations:** 1 Medical School of Chinese People’s Liberation Army General Hospital, Beijing, China; 2 Pharmaceutical Sciences Research Division, Department of Pharmacy, Medical Supplies Center, Chinese People’s Liberation Army General Hospital, Beijing, China

**Keywords:** cellular immunity, DDA cationic liposomes, humoral immunity, mucosal vaccine, pulmonary immunization, tissue-resident memory T cells (TRM cells)

## Abstract

**Introduction:**

Current injectable vaccines targeting respiratory viruses are highly effective in inducing systemic immunity, yet they fall short in establishing effective defenses at mucosal sites, ultimately failing to completely block viral infection and transmission. Developing novel immune strategies that can simultaneously activate mucosal and systemic immunity is vitally important.

**Methods:**

In this study, we created a pulmonary mucosal immune nanovaccine platform based on dimethyldioctadecyl ammonium (DDA) cationic liposomes. The immune effect was systematically evaluated using *in vitro* dendritic cell activation tests and a mouse pulmonary mucosal immune model.

**Results:**

The results showed that the prepared formulation had a uniform particle size (approximately 101 nm), positive zeta potential (+42.67 mV), and high encapsulation efficiency (over 80%). The formulation effectively stimulated the maturation of dendritic cells, induced high levels of antigen-specific IgG antibodies in serum and IgA antibodies in bronchoalveolar lavage fluid. It also significantly promoted the proliferation of activated T/B cells and the formation of tissue-resident memory T cells in lung tissues. Mechanistic studies revealed that the vaccine was retained in the lung for a long time and was subsequently detected in mediastinal lymph nodes, accompanied by increased activation-associated dendritic-cell phenotypes, thereby synergistically activating mucosal and systemic immune responses.

**Discussion:**

Overall, this study provides preliminary proof-of-concept evidence that the DDA liposome-based formulation has immunogenic potential following pulmonary mucosal administration. However, additional *in vivo* control groups and more rigorous validation of antigen-specific immune responses will be required in future studies.

## 1 Introduction

Respiratory infectious diseases, caused by various pathogens such as influenza virus, respiratory syncytial virus (RSV) and SARS-CoV-2, continue to pose a major global public health burden. Although vaccines targeting specific pathogens have shown remarkable effectiveness in controlling severe cases and reducing mortality rates, they often struggle to establish an effective immune response at the primary gateway of invasion, the respiratory mucosa. This often results in the inability to completely block asymptomatic infections and virus transmission (1). Consequently, the development of vaccine strategies that can induce strong mucosal immunity has emerged as a major frontier and challenge in the field of vaccinology.

The mucosal immune system comprises mucosal tissues, resident and recruited immune cells, and soluble effector molecules, including cytokines, chemokines, and antibodies (2). Among its key protective components are secretory IgA and tissue-resident memory T (TRM) cells, which contribute to rapid immune defense at sites of pathogen entry (3–5). In the lung mucosal immune environment, antigen-presenting cells play central roles in antigen sampling and initiation of adaptive immune responses. Dendritic cells (DCs) within it can either activate the immune response locally or migrate to the drainage lymph nodes to initiate systemic immunity after taking up antigens (6–8). Therefore, vaccine delivery through the respiratory mucosa is considered a promising strategy to better mimic natural exposure and promote both local and systemic immunity (9, 10).

However, mucosal vaccines still face numerous challenges. For example, mucosal physical barriers, such as the mucociliary clearance system, hinder antigen retention and uptake, leading to antigen removal and degradation before reaching immune cells (11). Moreover, identifying safe mucosal adjuvants remains a significant obstacle, as most candidate molecules pose toxicity risks (1). It is therefore urgently necessary to develop a delivery platform that enhances antigen stability, promotes the uptake by antigen-presenting cells (APCs), and safely strengthens the immune response.

Lipid-based nanoparticles, particularly cationic liposomes, have attracted increasing interest as mucosal vaccine delivery systems because they can improve antigen stability, promote interaction with mucosal surfaces, and facilitate uptake by APCs (12–16). They offer good biocompatibility and can enhance mucosal adhesion and cellular uptake through surface engineering modifications (17–20). Among these, cationic lipids such as DDA effectively adsorb onto negatively charged mucosal surfaces and the cell membranes of APCs through electrostatic interactions. This promotes antigen uptake and presentation (21, 22), thereby proving highly promising as vaccine adjuvants and carriers (23). Additionally, the Toll-like receptor 9 (TLR9) agonist CpG ODN, used as an adjuvant, can strongly promote the maturation of DCs and Th1-type immune responses. Its synergistic effect with cationic carriers has been confirmed in multiple studies (24, 25). Based on these considerations, we designed a pulmonary mucosal immunization platform combining DDA cationic liposomes with CpG ODN as an adjuvant. In this study, intratracheal administration was used as a controlled preclinical model of pulmonary delivery to achieve reproducible deposition of the formulation into the lower respiratory tract, as a proof-of-concept step toward the future development of an inhalable or nebulized vaccine formulation. A SARS-CoV-2 spike-derived branched peptide antigen based on the S_450–471_ region was used as the model antigen. We characterized the physicochemical properties of the formulation and evaluated its ability to induce mucosal, humoral, and cellular immune responses, as well as its preliminary *in vivo* biodistribution and immune-activation features. To our knowledge, studies integrating a DDA cationic liposome platform with a SARS-CoV-2 spike-derived branched peptide antigen for pulmonary mucosal immunization remain limited.

## 2 Materials and methods

### 2.1 Preparation of S_450-471_@DDA-Lipo

The peptide antigen used in this study was a branched SARS-CoV-2 spike-derived peptide designed on the basis of the S_450–471_ region (NYLYRLFRKSNLKPFERDISTE). This design incorporated the linear B-cell epitope S_455-470_ (LFRKSNLKPFERDIST) and the cytotoxic T-lymphocyte epitope S_462-471_ (KPFERDIST), which had been identified by our group through integrated bioinformatics-based prediction of antigenicity and immunogenic potential in conserved regions of the SARS-CoV-2 spike protein (26). The peptide antigen was synthesized by Chengdu Shengnuo Biopharmaceutical Co., Ltd.

S_450-471_@DDA-Lipo was prepared using a modified thin-film dispersion/hydration method (17, 22, 25). Briefly, 25 mg of 1,2-distearoyl-sn-glycero-3-phosphocholine (DSPC) (Macklin, Shanghai, China) and 5 mg of dimethyldioctadecylammonium (DDA) (Macklin, Shanghai, China) were dissolved in 3 mL dichloromethane/absolute ethanol (9:1, v/v). Based on the amounts used, the lipid composition corresponded to approximately 80 mol% DSPC and 20 mol% DDA. The organic solvent was removed by rotary evaporation at 45 °C to form a uniform lipid film. Subsequently, the lipid film was hydrated with 1 mL peptide aqueous solution containing 5 mg peptide antigen to yield the final liposomal suspension. No extrusion step was performed. Instead, the resulting suspension was subjected to ultrasonic dispersion and homogenization under an ice bath using an ultrasonic separator to obtain the final S_450-471_@DDA-Lipo formulation. The adjuvant used in this study was CpG ODN 1018, a class B CpG ODN (InvivoGen, San Diego, CA, USA; cat. vac-1018-1), with the sequence 5′-TGACTGTGAACGTTCGAGATGA-3′. CpG ODN 1018 was not encapsulated into the liposomal vesicles during formulation preparation. Instead, it was freshly mixed with the S_450-471_@DDA-Lipo suspension before *in vitro* stimulation or *in vivo* administration. The amount of CpG used in each experiment is specified in the corresponding subsections below.

### 2.2 Characterization of S_450-471_@DDA-Lipo

The particle size, polydispersity index (PDI), and zeta potential of S_450-471_@DDA-Lipo were measured using a Zetasizer Nano ZS laser particle size analyzer (Malvern, Worcestershire, UK). The particle size distribution shown in the manuscript was recorded in intensity mode, and the corresponding PDI was reported by the instrument software. For zeta potential measurement, samples were diluted 10-fold in distilled water prior to analysis. The diluted samples were measured at 25 °C and pH 7.0. The morphology of S_450-471_@DDA-Lipo was observed by transmission electron microscopy using a HT7800 transmission electron microscope (Hitachi, Tokyo, Japan).

Encapsulation efficiency was determined after separation of unencapsulated free peptide by ultrafiltration centrifugation using a Pierce™ ultrafiltration tube (PES 30K MWCO) (Thermo Fisher Scientific, Waltham, USA). The peptide concentration in the filtrate was measured using a BCA protein assay kit (YangGuangBio, Beijing, China). Encapsulation efficiency was calculated according to the following equation:


$$Encapsulation\;efficiency(\%)=\frac{total\;peptide\;input-free\;peptide\;in\;filtrate}{total\;peptide\;input}\times 100$$

In the present formulation, the lipid film was finally hydrated with 1 mL peptide aqueous solution containing 5 mg peptide antigen after complete removal of the organic solvent by rotary evaporation. Therefore, the initial peptide concentration in the final S_450-471_@DDA-Lipo suspension was 5 mg/mL. Based on the measured encapsulation efficiency (>80%), the final encapsulated peptide concentration was estimated to be approximately 4 mg/mL. Because particle number was not directly measured in the present study, the average antigen loading per liposome vesicle was not experimentally determined. More accurate quantification will require direct particle counting combined with antigen quantification in future studies.

For the stability study, freshly prepared S_450-471_@DDA-Lipo was stored at 4 °C, and the particle size and zeta potential were monitored at different time points during storage to evaluate physicochemical stability. *In vitro* antigen release behavior was not evaluated in the present study. This is a limitation of the current work, and antigen release kinetics will be investigated in future studies to further assess formulation stability and release behavior.

### 2.3 *In vitro* activation of bone marrow-derived dendritic cell and bone marrow-derived macrophage

BMDCs were generated from femurs and tibias of 8-week-old female BALB/c mice. After red blood cell lysis and washing, bone marrow cells were cultured in RPMI 1640 complete medium supplemented with 20 ng/mL IL-4 and 20 ng/mL granulocyte-macrophage colony-stimulating factor (GM-CSF) for 7 days to obtain BMDCs.

Bone marrow cells were isolated from the femurs and tibias of 8-week-old female BALB/c mice under sterile conditions. After red blood cell lysis and washing, the cells were cultured in RPMI 1640 complete medium supplemented with M-CSF at 20 ng/mL for 7 days to induce macrophage differentiation. The differentiated macrophages were then collected and used for the *in vitro* stimulation experiments.

For stimulation assays, BMDCs or BMDMs were seeded in 6-well plates at 2.5×10^5^ cells/well (cells were cultured in 2 mL complete medium per well) and incubated with different formulations for 24 h at 37 °C in 5% CO_2_. The treatment groups included PBS control, soluble peptide antigen (S_450-471_) alone (10 μg/well, final concentration 5 μg/mL), soluble CpG ODN alone (5 μg/well, final concentration 2.5 μg/mL), and the S_450-471_@DDA-Lipo+CpG (containing 10 μg peptide and 5 μg CpG per well, corresponding to final concentrations of 5 μg/mL peptide and 2.5 μg/mL CpG, respectively).

After stimulation, BMDCs and BMDMs were collected and stained for flow cytometric analysis. For BMDC/BMDM analysis, the following antibodies were used: APC anti-mouse CD11c (clone N418, cat. 117324, BioLegend, San Diego, CA, USA), PE anti-mouse CD80 (clone 16-10A1, cat. 104707, BioLegend), APC/Cyanine7 anti-mouse CD86 (clone GL-1, cat. 105030, BioLegend), Brilliant Violet 421 anti-mouse I-A/I-E (clone M5/114.15.2, cat. 107632, BioLegend), and FITC anti-mouse F4/80 (clone BM8, cat. 123108, BioLegend). All antibodies were incubated with the cells for 30 min at 4 °C in the dark, followed by washing and flow cytometric analysis. In addition, IFN-γ levels in the BMDC culture supernatants were measured using a mouse IFN-γ ELISA kit (Lianke Biotech, Hangzhou, China; cat. EK274) according to the manufacturer’s instructions. Absorbance was read at 450 nm using a microplate reader.

### 2.4 BMDCs viability assay

The viability of BMDCs after treatment with different formulations was evaluated using a CCK-8 assay. BMDCs were seeded in 96-well plates at 1×10^4^ cells/well in 100 μL complete medium per well. The treatment groups were set as follows: blank group, untreated control group, soluble peptide antigen (S_450-471_) group (0.5 μg/well, final concentration 5 μg/mL), soluble CpG ODN group (0.25 μg/well, final concentration 2.5 μg/mL), empty DDA-Lipo group (3 μg total lipid/well, final lipid concentration 30 μg/mL), and empty DDA-Lipo+CpG group (3 μg total lipid/well plus 0.25 μg CpG/well, corresponding to final concentrations of 30 μg/mL lipid and 2.5 μg/mL CpG, respectively). After 24 h of incubation with the indicated treatments, 10 μL CCK-8 solution was added to each well and the cells were further incubated for 1 h. The absorbance at 450 nm was then measured using a microplate reader, and cell viability was calculated accordingly.

### 2.5 *In vivo* immune effect of S_450-471_@DDA-Lipo

#### 2.5.1 Animals

Female Balb/c mice, aged 10–12 weeks, were used in the animal experiments. All mice were acquired from Beijing Sibeifu Biotechnology Co.,Ltd. The mice were housed at the Experimental Animal Center (SPF level) of the General Hospital of the Chinese PLA. This experimental protocol was approved by the Animal Experiment Ethics Committee of the General Hospital of the PLA. The approval number is 2023-X19-79.

#### 2.5.2 Immunization method

Nasal mucosal immunization method: Experimental mice are anesthetized by an intraperitoneal injection of pentobarbital sodium. After the mice are anesthetized, one is held in the left hand, with the thumb and index finger securing the head and neck to fully expose the nostrils. The mouse’s head is then tilted upward to facilitate the smooth flow of the formulation into the nasal cavity. Using a 1 mL insulin syringe preloaded with the formulation, the liposomal formulation is carefully dripped onto the mouse’s nostrils. The dripping speed should be as slow as possible, adhering to the principle of administering multiple small-volume drops. It is important to wait until each drop has fully entered the nostril before proceeding to the next, continuing this process until all the formulation has been administered. After the intranasal administration, the mouse should be maintained in the same posture for at least 30 seconds to prevent the solution from flowing out. Meanwhile, the mouse’s reaction should be closely observed to ensure that there are no abnormal symptoms, such as dyspnea.
Intratracheal immunization via orotracheal route: First, disinfect a mouse tracheal intubation needle (27G blunt needle) using 75% medical alcohol and then load an appropriate amount of formulation into a syringe for later use. Anesthetizing the mouse via intraperitoneal injection with pentobarbital sodium. Place the mouse in a supine position and secure it on a tracheal intubation operating table, ensuring the upper incisors are hooked onto a steel wire at the top of the table. Adjust the posture to ensure the body is straight and level, aligning the head, neck, and body. Perform the operate from the head side of the mouse. Hold a cold-light laryngoscope in your left hand, insert it into the mouse’s mouth, and press down on the tongue and lower jaw to open the oral cavity, as widely as possible, thereby exposing the field of view. The tracheal opening in mice is positioned relatively close to the base of the tongue. Using the cold light of the laryngoscope, identify the tracheal opening and the location of the vocal cords. Note that the vocal cords open and close transversely with respiration. Hold the syringe for tracheal intubation in your right hand. When the vocal cords open, gently insert the intubation needle into the mouse’s trachea. As the needle tip contacts the tracheal cartilaginous rings, continue to advance the needle slowly for about 1-1.5 cm deeper into the trachea until it reaches the hilum of the lung, thereby completing the tracheal intubation procedure. Confirm visually that the intubation needle is positioned between the vocal cords. Slowly inject the formulation from the syringe into the mouse’s lung, taking care to avoid inducing a cough. After administering the formulation, carefully withdraw the intubation needle. Maintain the mouse in the same intubation posture for over 30 seconds to prevent the formulation from being expelled through coughing, and monitor the mouse for any abnormal signs such as dyspnea. Following administration, remove the mouse from the table and auscultate for moist rales in its lung to further confirm successful mucosal administration to the lungs.

#### 2.5.3 Collection of nasal and bronchoalveolar lavage fluid

Collection of nasal lavage fluid (NLF): After euthanizing the mouse by cervical dislocation, place the mouse in a supine position on a dissection table. Insert scissors into the mouse’s oral cavity, cut through the cheeks on both sides, and separate the lower jaw. Lift the lower jaw, tongue and other soft tissues of the throat and neck to expose the hard palate. The posterior nasal passage, a duct within the soft palate, will become visible. This passage is located directly above the tracheal inlet, near the base of the tongue, and can be used to identify the posterior nasal passage after dissection. Hold the mouse with the left hand, keeping the head pointed downward. With the right hand, use a 1 mL syringe preloaded with 500 μL of ice-cold PBS (the needle tip should be pre-cut to be flat). Insert the needle about 0.5 cm into the opening of the posterior nasal passage. Slowly inject the PBS and collect the lavage fluid from the nostrils using a 1.5 mL centrifuge tube. It is possible to collect approximately 500 μL of NLF. Centrifuge the collected fluid at 4000 rpm for 15 minutes. Collect the supernatant, aliquot it, and store it at -20 °C for later use.
Collection of bronchoalveolar lavage fluid (BALF): After euthanizing the mouse by cervical dislocation, place the mouse in a supine position on a dissection table. Disinfect the neck skin with 75% medical alcohol. Use surgical scissors to make a midline incision about 2 cm long along the neck to expose the trachea. Carefully separate the overlying tissue and the muscles surrounding the trachea. Using ophthalmic scissors, make a small incision between two cartilage rings near the cephalic end of the trachea. Carefully insert a sterile gavage needle through this incision into the trachea.Fasten a surgical suture around the trachea about 0.5 cm from the incision toward the lungs, and tie both the trachea and the gavage needle together to prevent backflow of the lavage fluid. Attach a 1 mL syringe (needle removed) preloaded with 500 μL of ice−cold PBS to the gavage needle. Slowly infuse the PBS into the lungs, gently massaging the thoracic cavity for about 20 seconds, and then, while rhythmically compressing the chest with the left hand, gradually withdraw the lavage fluid with the right hand. Be careful to avoid excessive negative pressure, which could damage lung tissue. Collect the lavage fluid in a 1.5 mL centrifuge tube. Repeat the procedure, and collect a total of approximately 500 μL of BALF. Centrifuge at 4000 rpm for 15 minutes. Collect the supernatant, aliquot it, and store it at -20 °C for later use.

#### 2.5.4 Detection of IgG after immunization with S_450-471_@DDA-Lipo+CpG by different mucosal routes

Fifteen mice were randomly divided into three groups: a control group, a nasal mucosal immunization group, and a pulmonary mucosal immunization group. Mice in the experimental groups were immunized on days 0 and 14 via either the nasal route or intratracheal (IT) immunization through the orotracheal route. Each mouse received a total volume of 42 μL, containing 200 μg of peptide antigen (from S_450-471_@DDA-Lipo stock at 5 mg/mL) and 10 μg of CpG ODN (from a 5 mg/mL stock). For the nasal immunization, the 42 μL volume was equally divided into two 21 μL portions and gently instilled dropwise into each nostril. For pulmonary immunization via the orotracheal route, the mice were anesthetized, and a sterile 27G blunt needle was inserted into the trachea through the oral cavity; the 42 μL formulation was then injected as a single bolus. After 42 days, serum samples were collected from the mice in each group to detect antigen-specific IgG. The NLF and BALF from the nasal and the pulmonary mucosal immunization groups were collected to detect antigen-specific IgA. The collection procedures for NLF and BALF are described in Section 2.5.3.

#### 2.5.5 Evaluation of the immune effect of S_450-471_@DDA-Lipo+CpG through pulmonary mucosa

Twenty mice were randomly divided into four groups: a control group (PBS), a pulmonary mucosal immunization group (Intratracheal, IT), a combined subcutaneous and pulmonary mucosal immunization group (SC+IT), and a subcutaneous immunization group (SC). The SC group was included as a conventional systemic immunization comparator to distinguish route-dependent effects of pulmonary mucosal immunization from those induced by a non-mucosal administration route, whereas the PBS group served as the negative control. The mice in the IT group received S_450-471_@DDA-Lipo+CpG via orotracheal administration on days 0, 14, and 28. The SC+IT group received a subcutaneous injection on day 0 followed by orotracheal immunizations on days 14 and 28. The SC group received subcutaneous injections on days 0, 14, and 28. For each administration route, the dose volume was 42 μL per mouse (containing 200 μg antigen and 10 μg CpG ODN). The orotracheal procedure is detailed in Section 2.5.2.

Forty-two days after the first immunization, serum, BALF, and the indicated tissues were collected for subsequent immunological analyses. The collection procedures for BALF are described in Section 2.5.3. Antigen-specific antibody detection was performed by ELISA as described in Section 2.5.6. Total IgA in BALF and serum cytokine levels were determined as described in Section 2.5.8.

#### 2.5.6 ELISA detection of antigen-specific antibody levels in serum and mucosal samples

Antigen-specific antibody titers in serum and mucosal samples were determined by indirect ELISA. Before use, all reagents were equilibrated to room temperature. Briefly, the peptide antigen was dissolved in distilled water at 2 mg/mL as a stock solution. The antigen stock was then diluted in coating buffer to a final concentration of 2 μg/mL. A total of 100 μL of the diluted antigen solution was added to each well of a 96-well ELISA plate (Corning, NY, USA; cat. 3364), and the plate was incubated overnight at 4 °C for coating.

On the following day, the coating solution was discarded and the plate was washed three times with washing buffer. For each wash, the wells were filled with washing buffer, allowed to stand for 3 min with gentle shaking, and then emptied and blotted dry on absorbent paper. The wells were then blocked with 200 μL BSA blocking buffer (YangGuangBio, Beijing, China; cat. C210225) per well at 37 °C for 2 h, followed by three washes.

Serum or mucosal samples were serially diluted in a two-fold gradient, and 100 μL of each diluted sample was added to the corresponding wells. After incubation at 37 °C for 1 h, the plate was washed three times. Subsequently, 100 μL of appropriately diluted HRP-conjugated secondary antibody was added to each well according to the manufacturer’s instructions, followed by incubation at 37 °C for 1 h and three additional washes. The following HRP-conjugated secondary antibodies were used: HRP anti-mouse IgG (Lianke Biotech, Hangzhou, China; cat. GAM007), HRP anti-mouse IgG1 (ABclonal, Wuhan, China; cat. AS066), HRP anti-mouse IgG2a (ABclonal, Wuhan, China; cat. AS065), and HRP anti-mouse IgA (Proteintech, Rosemont, IL, USA; cat. SA00012-7).

For color development, 100 μL TMB single-component substrate solution (YangGuangBio, Beijing, China; cat. P223780) was added to each well and incubated at 37 °C in the dark for 10–30 min. The reaction was stopped by adding 50 μL stop solution (YangGuangBio, Beijing, China; cat. P223723) to each well. The absorbance at 450 nm was measured using a microplate reader. Antibody titers were determined from the ELISA dilution curves. Antigen-specific IgG, IgG1, and IgG2a in serum, as well as antigen-specific IgA in BALF and/or NLF, were determined using the corresponding secondary antibodies.

#### 2.5.7 Flow cytometric analysis of spleen and lung lymphocytes after ex vivo peptide restimulation

Forty-two days after the first immunization, spleen and lung tissues were collected from mice and processed into single-cell suspensions for flow cytometric analysis. Spleens and Lungs were mechanically dissociated to prepare single-cell suspensions. For ex vivo peptide restimulation, 1×10^6^ cells from each sample were incubated with the S_450–471_ peptide at a final concentration of 2 μg/mL for 24 h at 37 °C in a humidified 5% CO2 atmosphere prior to surface staining.

For splenic lymphocyte analysis, cells were stained with APC anti-mouse CD3 (clone 17A2, cat. 100236, BioLegend), VioBlue anti-mouse CD45R(B220) (clone REA755, cat. 130-110-851, Miltenyi Biotec, Bergisch Gladbach, Germany), FITC anti-mouse CD4 (clone GK1.5, cat. 100406, BioLegend), PE anti-mouse CD8a (clone S18018E, cat. 162304, BioLegend), APC-Vio770 anti-mouse CD44 (clone IM7.8.1, cat. 130-124-708, Miltenyi Biotec), and PE-Vio770 anti-mouse CD62L (clone REA828, cat. 130-112-649, Miltenyi Biotec).

For lung lymphocyte analysis, cells were stained with APC anti-mouse CD3 (clone 17A2, cat. 100236, BioLegend), VioBlue anti-mouse CD45R(B220) (clone REA755, cat. 130-110-851, Miltenyi Biotec), FITC anti-mouse CD4 (clone GK1.5, cat. 100406, BioLegend), PE anti-mouse CD8a (clone S18018E, cat. 162304, BioLegend), APC-Vio770 anti-mouse CD44 (clone IM7.8.1, cat. 130-124-708, Miltenyi Biotec), VioGreen anti-mouse CD69 (clone H1.2F3, cat. 130-103-952, Miltenyi Biotec), PE/Cyanine7 anti-mouse CD11a (clone 2D7, cat. 162914, BioLegend), and PE/Dazzle 594 anti-mouse CD103 (clone QA17A24, cat. 156910, BioLegend). All antibodies were incubated with the cells for 30 min at 4 °C in the dark, followed by washing and flow cytometric analysis. Representative gating strategies are provided in the **Supplementary Figures**.

#### 2.5.8 Determination of total IgA in BALF and serum cytokine levels

The total IgA concentration in BALF was determined using a mouse IgA ELISA kit (Lianke Biotech, Hangzhou, China; cat. EK274) according to the manufacturer’s instructions. Briefly, BALF samples were thawed on ice and added to the pre-coated ELISA plate together with the provided standards. After incubation and washing, the corresponding HRP-conjugated detection reagent was added, followed by color development with substrate solution. The reaction was terminated with stop solution, and the absorbance at 450 nm was measured using a microplate reader. Total IgA concentrations were calculated from the standard curve and expressed as ng/mL.

Serum concentrations of IFN-γ (Lianke Biotech, Hangzhou, China; cat. EK274), granzyme B (Lianke Biotech, Hangzhou, China; cat. EK2173), IL-4 (Lianke Biotech, Hangzhou, China; cat. EK204), and IL-10 (Lianke Biotech, Hangzhou, China; cat. EK210) were measured using commercial mouse ELISA kits according to the manufacturers’ instructions. Briefly, serum samples and standards were added to the corresponding ELISA plates, incubated, washed, and then reacted with the appropriate detection reagents. After color development and reaction termination, absorbance was read at 450 nm using a microplate reader. Cytokine concentrations were calculated from the respective standard curves.

#### 2.5.9 *In vivo* biodistribution of fluorescently labeled DDA-Lipo

To evaluate the *in vivo* biodistribution of the liposomal formulation after pulmonary mucosal administration, DDA-Lipo was labeled with DiR (Uelandy, Suzhou, China). Mice were administered the fluorescently labeled formulation via the orotracheal route as described in Section 2.5.2. *In vivo* fluorescence imaging was performed at 0, 1, 2, 3, 6, 9, 10 days after administration using an IVIS Spectrum CT (PerkinElmer, Waltham, USA). At the indicated endpoint, mice were euthanized and major organs, including the lung, heart, liver, spleen, kidney, cervical lymph nodes, axillary lymph nodes, and mediastinal lymph nodes, were isolated for ex vivo imaging and fluorescence quantification.

### 2.6 Statistical analysis

**Figure 1:**
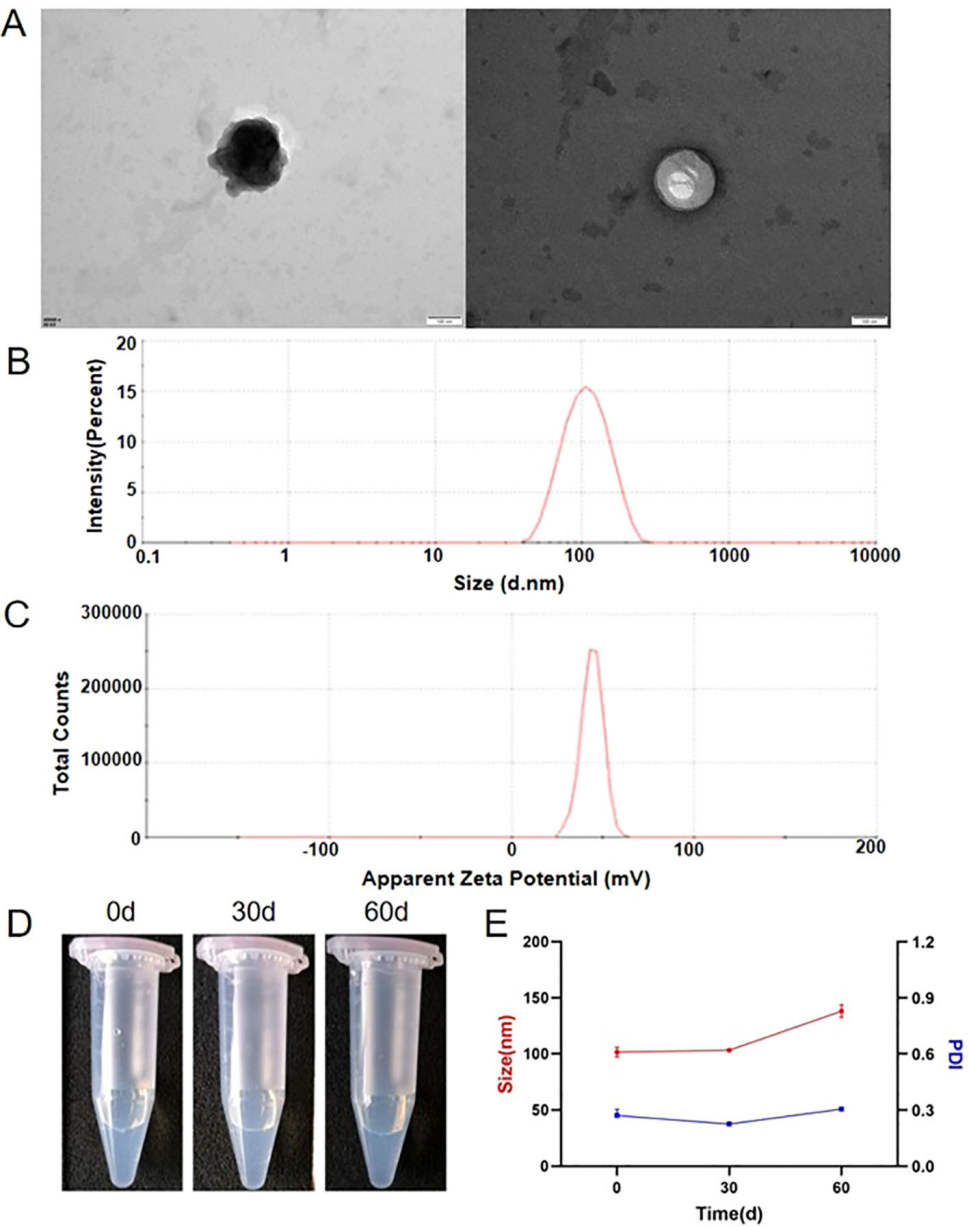
Physicochemical characterization of S_450-471_@DDA-Lipo. **(A)** Transmission electron microscopy image of S_450-471_@DDA-Lipo. **(B)** Particle size distribution of S_450-471_@DDA-Lipo. **(C)** Zeta potential of S_450-471_@DDA-Lipo. **(D, E)** Changes in particle size and zeta potential during storage at 4 °C. Data are presented as mean ± SD. Scale bar = 100 nm.

**Figure 2:**
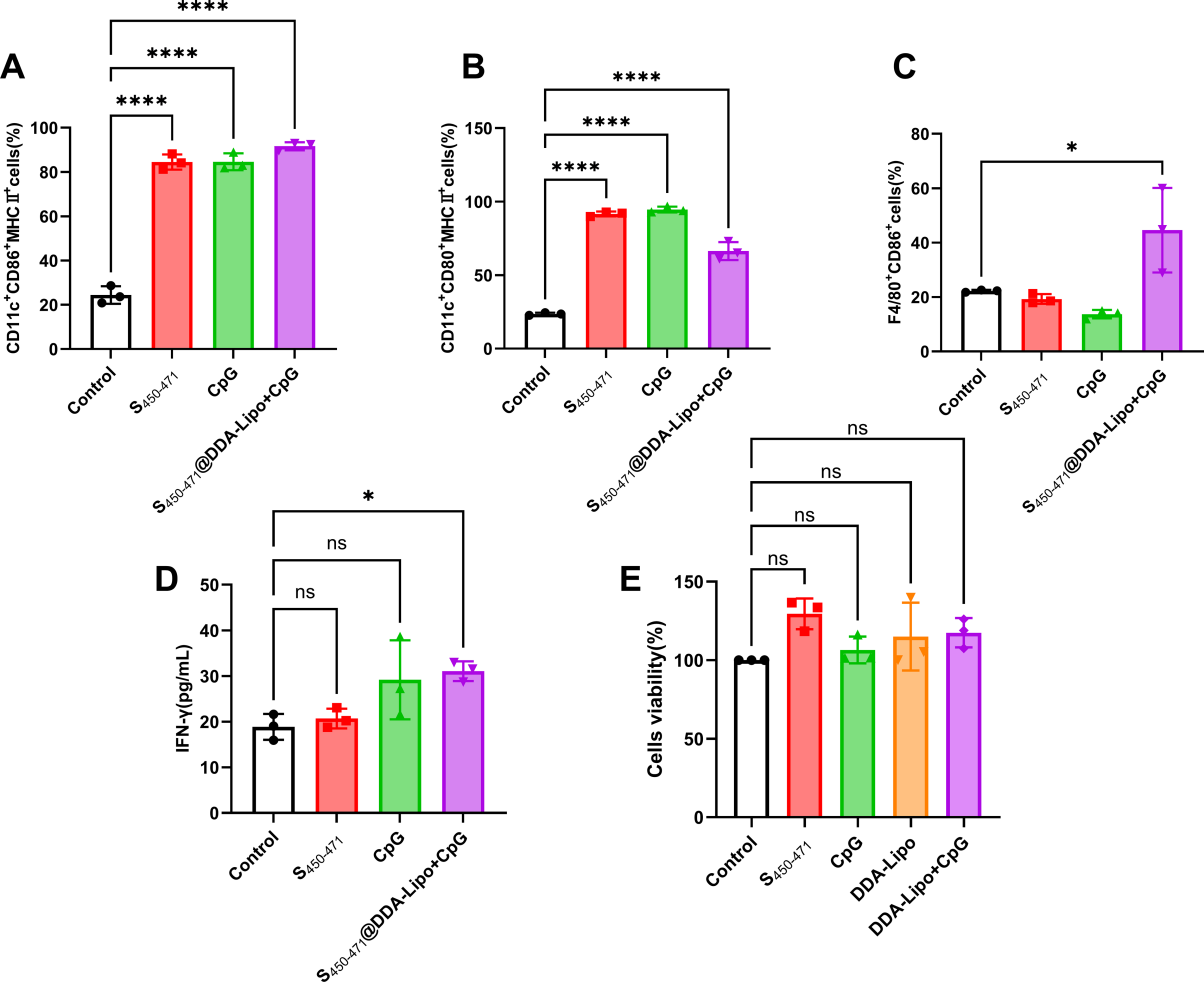
Expression of co-stimulatory molecules and MHC II on BMDCs after treatment with different formulations. **(A)** CD86+MHC II+ BMDCs; **(B)** CD80+MHC II+ BMDCs; **(C)** CD86+ expression in BMDMs; **(D)** IFN-γ levels in BMDC culture supernatants; **(E)** BMDC viability assessed by CCK-8 assay. The treatment groups included PBS, soluble peptide antigen (S450-471), soluble CpG ODN, and S_450-471_@DDA-Lipo+CpG. N = 3 per group. Statistical analysis was performed by one-way ANOVA followed by Dunnett's multiple comparisons test versus the control group. *P < 0.05; **P < 0.01; ***P < 0.001; ****P < 0.0001. ns, not significant.

All data are presented as mean ± SD and were analyzed using GraphPad Prism 10. Statistical differences among multiple groups were evaluated by one-way analysis of variance (ANOVA) followed by the appropriate *post hoc* multiple-comparisons test. In most analyses, Tukey’s multiple comparisons test was used, whereas Dunnett’s multiple comparisons test was applied for the analyses shown in **Figures 1** and **2**, in which each experimental group was compared with the control group. Comparisons between two groups were performed using Student’s t-test. Statistical significance was denoted as *P < 0.05, **P < 0.01, ***P < 0.001, ****P < 0.0001, ns, (not significant).

## 3 Results

### 3.1 Preparation and characterization of S_450-471_@DDA-Lipo

**Figure 3:**
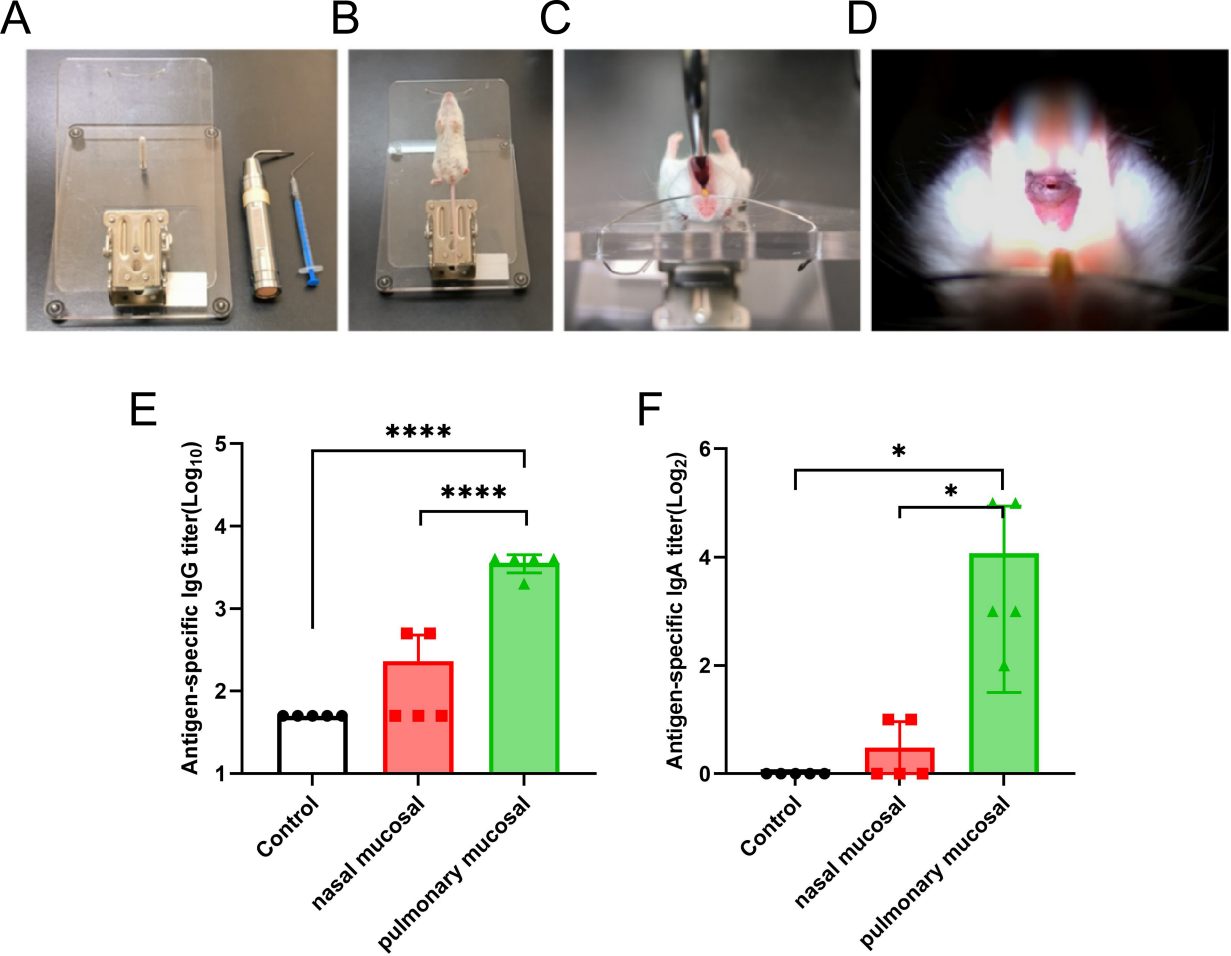
Orotracheal pulmonary immunization procedure and antibody responses. **(A)** Schematic illustration of the orotracheal intubation setup. **(B)** Mouse positioned on the intubation platform with upper incisors fixed. **(C)** Cold_light laryngoscope inserted into the oral cavity to depress the tongue and expose the glottis. **(D)** Visualization of the vocal cords (open during inspiration) and Intubation with a 27G blunt needle advanced through the vocal cords into the trachea. **(E, F)** Antigen_specific IgG titers in serum and IgA titers in BALF after nasal or pulmonary immunization. Mice were immunized with S_450-471_@DDA_Lipo+CpG via either the nasal route or the pulmonary route. N=5 per group. Statistical analysis was performed by one-way ANOVA followed by Tukey's multiple comparisons test unless otherwise indicated. *P<0.05; **P<0.01; ***P<0.001; ****P < 0.0001.

A DDA-based liposome capable of immunization through the respiratory mucosa was constructed using the thin film dispersion method. Previous studies have shown that the small particle size (approximately 100 nm) of liposomes enhances their penetration into the mucosa and their uptake by macrophages and DCs in the mucosal epithelial layer. The positive surface charge of cationic liposomes has been reported to favor interaction with negatively charged mucosal surfaces and immune cells, and may be associated with prolonged mucosal retention and enhanced cellular uptake (27–30). The prepared S_450-471_@DDA-Lipo showed regular morphology and spherical shape under the transmission electron microscope (**Figure 1A**). Its particle size was about 101.6 ± 4.16 nm, the PDI was 0.271 ± 0.032, zeta potential was +42.67 ± 3.50 mV (**Figures 3B, C**), particle size distribution was uniform, and the encapsulation efficiency was above 80% as determined by BCA method. In addition, the storage stability of S_450-471_@DDA-Lipo was evaluated at 4 °C by monitoring changes in particle size and zeta potential over time (**Figures 3D, E**). The formulation remained relatively stable during the first 30 days of storage. After 60 days, the particle size increased from 101.58 nm to 138.1 nm, indicating partial size growth during prolonged storage; however, the formulation still remained within the nanoscale range considered acceptable for respiratory mucosal delivery.

### 3.2 S_450-471_@DDA-Lipo+CpG can effectively stimulate the activation of dendritic cells

Since dendritic cells (DCs) are the key initiators of adaptive immunity, we first evaluated whether the different formulations could promote BMDC maturation *in vitro*. As shown in **Figures 2A, B**, compared with the PBS group, treatment with the tested formulations increased the expression of the activation markers CD80, CD86, and MHC II on BMDCs, indicating enhanced dendritic cell maturation.

We also evaluated the effect of the formulations on macrophages. As shown in **Figure 2C**, CD86 expression in macrophages was significantly increased in the S_450-471_@DDA-Lipo+CpG group compared with the PBS group.

We further measured IFN-γ levels in the BMDC culture supernatants as a supplementary immune-related readout. As shown in **Figure 2D**, only the S_450-471_@DDA-Lipo+CpG group induced a statistically significant increase in IFN-γ secretion compared with the PBS group (P < 0.05), whereas the soluble peptide group and the soluble CpG group did not show significant differences (P > 0.05). Because IFN-γ is not a classical marker for dendritic cell activation, this result was interpreted only as an auxiliary immune-related observation and not as the primary basis for evaluating BMDC maturation.

BMDC viability under the tested formulation conditions was evaluated by CCK-8 assay (**Figure 2E**). No detectable cytotoxicity was observed at the tested concentration, indicating that the observed changes in activation-marker expression were not attributable to overt cell toxicity. However, dose-dependent toxicity and uptake optimization studies were not performed in the present work and will require further investigation in future studies.

### 3.3 The antibody response induced by pulmonary mucosal immunization with S_450-471_@DDA-Lipo+CpG was stronger than that induced by nasal mucosal immunization

To explore the most suitable route for respiratory mucosal inoculation using S_450-471_@DDA-Lipo+CpG, we compared the antibody response levels in mice following nasal and pulmonary mucosal inoculations. The steps involved pulmonary mucosal immunity are depicted in **Figures 3A–D**. The results, as shown in **Figures 3E, F**, indicated that the nasal mucosal immunization group exhibited low levels of antigen-specific IgG and IgA antibody responses. Conversely, the pulmonary mucosal immunization group induced significant antibody responses in all mice: The pulmonary mucosal immunization group showed significantly higher serum antigen-specific IgG titers and BALF antigen-specific IgA titers than the nasal mucosal immunization group (3600 ± 894.4, *P* < 0.0001 for IgG; 16.80 ± 13.97, P < 0.05 for IgA). Statistical analysis revealed that compared to nasal mucosal immunization, pulmonary mucosal immunization significantly enhanced the production of systemic IgG antibodies and local mucosal IgA antibodies, with a considerable higher intensity of response. These results suggest that S_450-471_@DDA-Lipo+CpG is more suitable for pulmonary mucosal immunization.

### 3.4 Adaptive immune responses

To evaluate the immunogenicity of S_450-471_@DDA-Lipo+CpG administered through different routes, we analyzed the adaptive immune responses in the lungs (mucosal site), spleen (systemic lymphoid organs), and serum. For flow cytometric analysis of splenic and pulmonary lymphocyte subsets, single-cell suspensions were subjected to ex vivo restimulation with the S_450–471_ peptide prior to staining. Mice were immunized endotracheally (IT, pulmonary mucosal), subcutaneously (SC, systemic), or with a combination of both (SC+IT).

#### 3.4.1 Mucosal immunity triggers superior local cellular and humoral immunity in the lung

**Figure 4:**
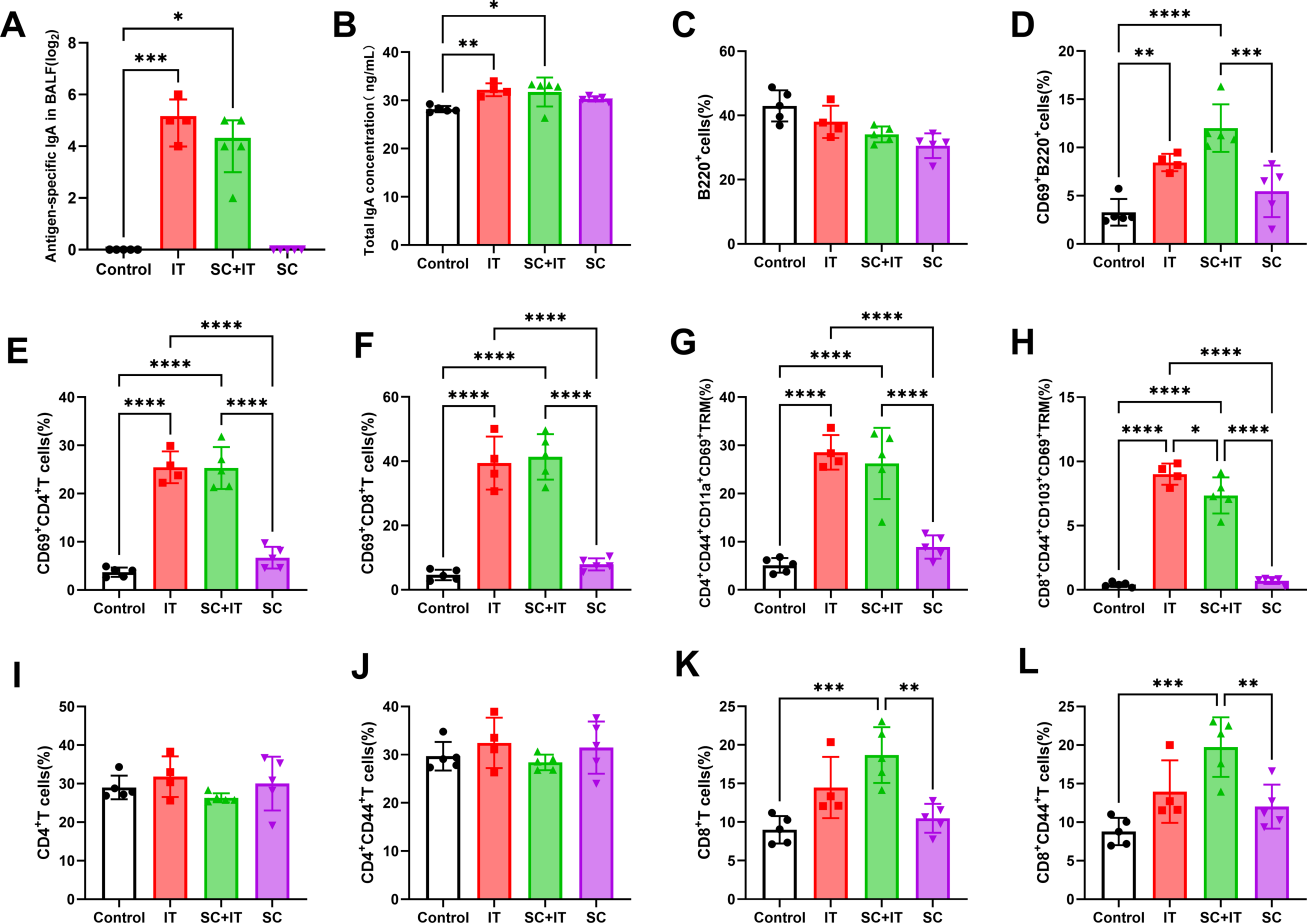
Local mucosal immune responses in the lung after immunization. **(A)** Antigen-specific IgA titer in BALF; **(B)** total IgA concentration in BALF; **(C)** proportion of B cells in the lung; **(D)** proportion of activated B cells in the lung; **(E)** proportion of activated CD4+ T cells in the lung; **(F)** proportion of activated CD8+ T cells in the lung; **(G)** proportion of CD4+ TRM-like cells in the lung; **(H)** proportion of CD8+ TRM-like cells in the lung; **(I)** proportion of CD4+ T cells in the lung; **(J)** proportion of CD4+ memory T cells in the lung; **(K)** proportion of CD8+ T cells in the lung; **(L)** proportion of CD8+ memory T cells in the lung. N = 5 per group. Statistical analysis was performed by one-way ANOVA followed by Tukey's multiple comparisons test unless otherwise indicated. *P < 0.05; **P < 0.01; ***P < 0.001; ****P < 0.0001.

To assess the immune response associated with the formulation at the primary portal of entry, we first analyzed cellular and humoral immunity in the lungs. As shown in **Figure 4A**, compared with the control group, the IT group showed a markedly increased antigen-specific IgA titer of 36 ± 20.13 (P < 0.001) and the SC+IT group showed an antigen-specific IgA titer of 20 ± 12 (P < 0.05). In contrast, total IgA concentration in BALF (**Figure 4B**) showed only modest differences among groups: The mean total IgA concentration in BALF was 28.38 ± 0.66 ng/mL in the control group, 32.23 ± 1.34 ng/mL in the IT group, 31.75 ± 3.00 ng/mL in the SC+IT group, and 30.24 ± 0.52 ng/mL in the SC group. These findings indicate that pulmonary mucosal immunization preferentially enhanced the antigen-specific IgA response rather than causing a proportional increase in the total mucosal IgA pool. To evaluate the cellular mechanisms that support this antibody response, we next examined lymphocyte activation in the lungs.

There was no significant difference in the proportion of B cells among the groups (**Figure 4C**). However, the proportion of activated B cells was significantly higher in both the IT group (P < 0.01) and the SC+IT group (P < 0.0001) than in the control and SC groups (**Figure 4D**). The increased proportion of activated B cells was consistent with enhanced local mucosal immune activation.

It should be noted that the T-cell populations shown in **Figure 4** were analyzed from lung-derived single-cell suspensions rather than from BALF. Following ex vivo restimulation with the S_450–471_ peptide, both the IT and SC+IT groups showed markedly increased proportions of activated CD4^+^ and CD8^+^ T cells in the lung (**Figures 4E, F**), as well as increased frequencies of CD4^+^ and CD8^+^ tissue-resident memory T (TRM) cells (**Figures 4G, H**), compared with the control and SC groups (P < 0.0001). No statistically significant differences were observed in the proportions of CD4^+^ T cells or CD4^+^ memory T cells between the IT group and the control group (**Figures 4I, J**). The SC+IT group showed significantly increased proportions of CD8^+^ T cells and CD8^+^ memory T cells compared with the control and SC groups (**Figures 4K, L**; P < 0.01). In contrast, the SC group did not induce significant pulmonary peptide-restimulated T-cell phenotypic changes under the present experimental conditions.

#### 3.4.2 Combined mucosal and subcutaneous immunization enhanced systemic humoral responses and induced a mixed Th1/Th2-associated cytokine profile

**Figure 5:**
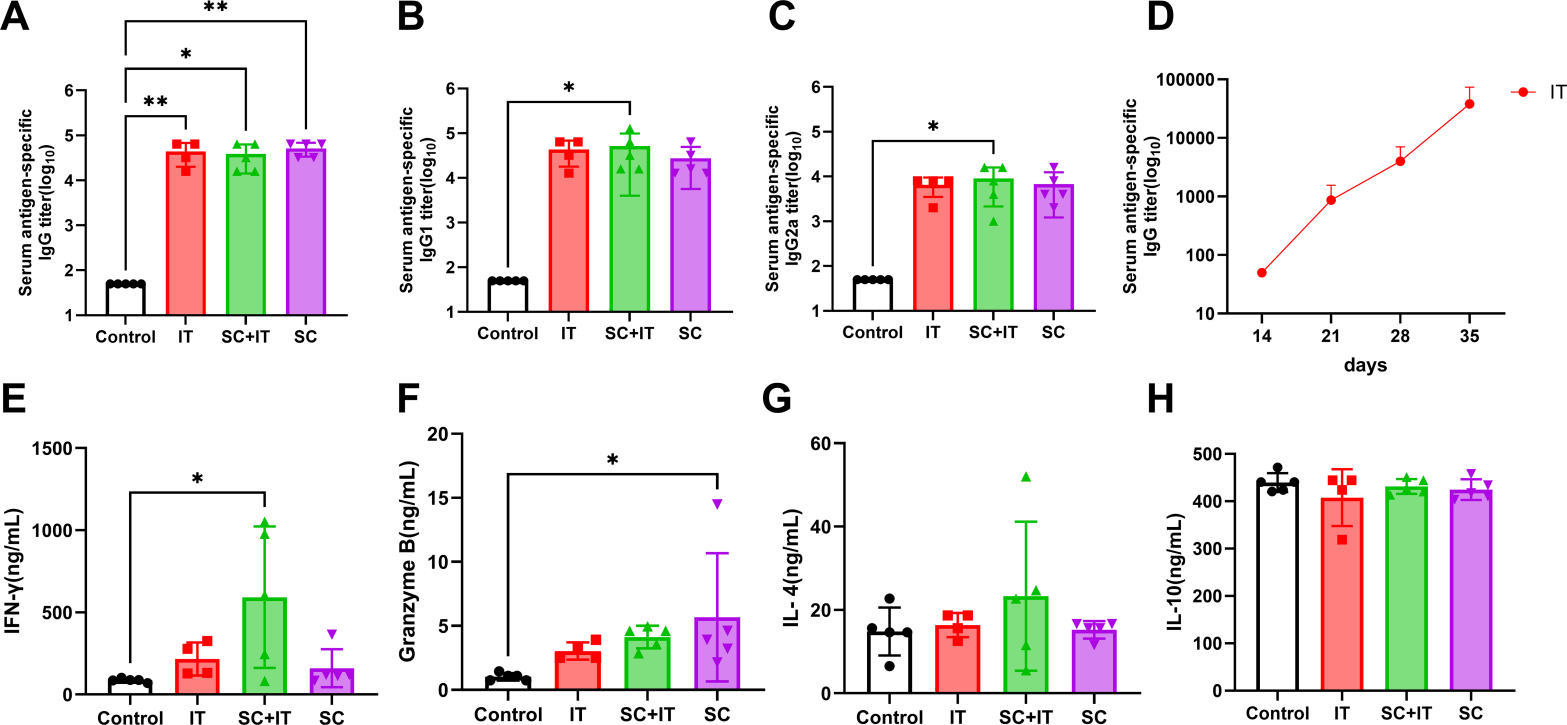
Systemic humoral immune responses and serum cytokine levels after immunization. **(A)** Antigen-specific IgG titer in serum; **(B)** antigen-specific IgG1 titer in serum; **(C)** antigen-specific IgG2a titer in serum; **(D)** time-course of serum antigen-specific IgG titer in the IT group only at different immunization time points; **(E)** serum IFN-γ level; **(F)** serum granzyme B level; **(G)** serum IL-4 level; **(H)** serum IL-10 level. N = 5 per group. Statistical analysis for panels A-C and E-H was performed by one-way ANOVA followed by Dunnett’s multiple comparisons test versus the control group. *P < 0.05; **P < 0.01; ***P < 0.001; ****P < 0.0001.

We assessed the systemic humoral immune response by measuring antigen-specific IgG titers in the serum and key cytokine secretion. Compared with the control group, the mean log_10_ antigen-specific IgG titers were 4.58 ± 0.29 (P < 0.01), 4.45 ± 0.26 (P < 0.05), and 4.69 ± 0.16 (P < 0.01) in the IT, SC+IT, and SC groups, respectively (**Figure 5A**). The mean log_10_ antigen-specific IgG1 titers were 4.56 ± 0.33 (P > 0.05), 4.56 ± 0.39 (P < 0.05), and 4.35 ± 0.31 (P > 0.05) in the IT, SC+IT, and SC groups, respectively (**Figure 5B**). Only the SC+IT group showed a statistically significant increase compared with the control group, whereas the IT and SC groups did not reach statistical significance. The mean log_10_ antigen-specific IgG2a titers were 3.75 ± 0.30 (P > 0.05), 3.78 ± 0.50 (P < 0.05), and 3.72 ± 0.34 (P > 0.05) in the IT, SC+IT, and SC groups, respectively (**Figure 5C**). **Figure 5D** showed the dynamic changes of antibodies in the IT group at different immunization time points. On days 14, 28 and 42, the mean log_10_ antigen-specific IgG titers in serum were 2.94 ± 0.25, 3.60 ± 0.24, and 4.58 ± 0.29, respectively.

The results of serum cytokine detection showed that the serum IFN-γ level of each group was higher than that of the control group. The average concentration of IFN-γ was 86.6 ± 11.34 ng/mL in control group, 215.9 ± 100.8 ng/mL (P > 0.05) in IT group, 592.4 ± 430.2 ng/mL (P < 0.05) in SC+IT group, and 159.7 ± 115.4 ng/mL (P > 0.05) in SC group. The increase was most significant in the SC+IT group (**Figure 5E**). In terms of Granzyme B, the concentration of the control group was 1.02 ± 0.29 ng/mL, and the IT group, SC+IT group and SC group increased to 3.03 ± 0.67 ng/mL (P > 0.05), 4.12 ± 0.88 ng/mL (P > 0.05) and 5.67 ± 5.00 ng/mL (P < 0.05), respectively, and the concentration of SC group was the highest (**Figure 5F**). The IL-4 concentration was 14.82 ± 5.73 ng/mL (P > 0.05) in the control group, 16.39 ± 2.90 ng/mL (P > 0.05) in the IT group, 23.31 ± 17.88 ng/mL (P > 0.05) in the SC+IT group, and 15.23 ± 2.10 ng/mL (P > 0.05) in the SC group, respectively, with no significant difference among the groups (**Figure 5G**). Similarly, the concentration of IL-10 was 439.4 ± 20.11 ng/mL in the control group, 407.8 ± 60.01 ng/mL (P > 0.05), 431.3 ± 15.77 ng/mL (P > 0.05), and 424.5 ± 21.73 ng/mL (P > 0.05) in the IT, SC+IT, and SC groups, respectively, and there was no statistically significant difference between the groups (**Figure 5H**). Taken together, these findings suggest a mixed Th1/Th2-associated systemic immune profile rather than a purely Th1-biased response.

#### 3.4.3 Peptide-restimulated splenic T-cell memory phenotypes following vaccination

**Figure 6:**
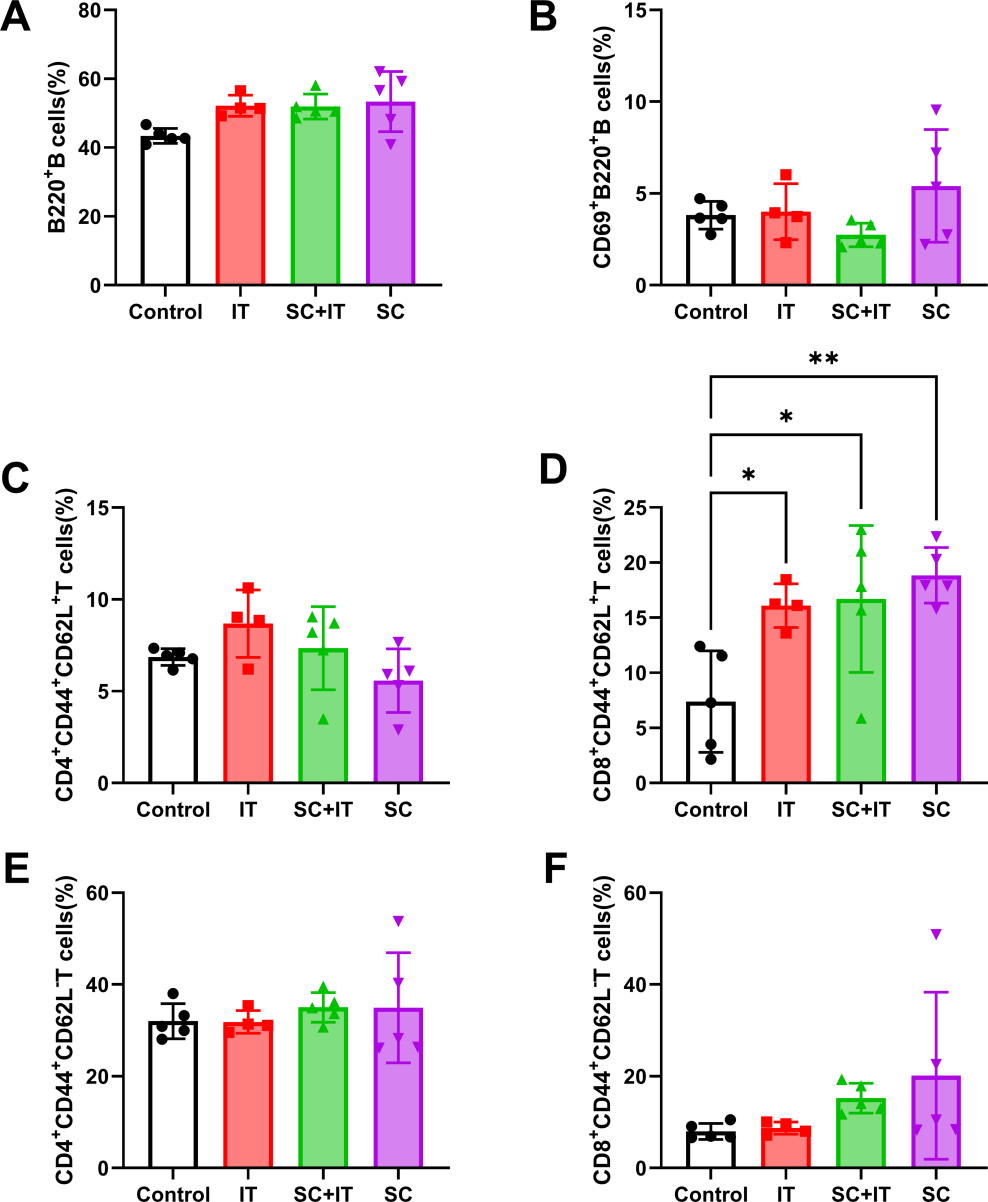
Peptide-restimulated splenic immune-cell phenotypes after vaccination. **(A)** proportion of B cells in the spleen; **(B)** proportion of activated B cells in the spleen; **(C)** proportion of CD4+ central memory T cells; **(D)** proportion of CD8+ central memory T cells; **(E)** proportion of CD4+ effector memory T cells; **(F)** proportion of CD8+ effector memory T cells. N = 5 per group. Statistical analysis was performed by one-way ANOVA followed by Tukey’s multiple comparisons test. *P < 0.05; **P < 0.01; ***P < 0.001; ****P < 0.0001.

In the spleen, no statistically significant differences in the proportion of total B cells were observed among the groups (**Figure 6A**). Likewise, the proportion of activated B cells did not differ significantly among groups (**Figure 6B**). Therefore, under the current experimental conditions, no definitive route-dependent effect on splenic B-cell expansion can be concluded. We then investigated how different routes of administration influence the generation of T-cell memory, which is crucial for long-term protection.

After ex vivo restimulation with the S_450–471_ peptide, no statistically significant differences were observed among the groups in the proportions of splenic central memory T cells (TCM) or effector memory T cells (TEM) (**Figures 6C–F**). Therefore, based on the current data, no route-dependent differences in splenic T-cell memory polarization can be concluded.

### 3.5 S_450-471_@DDA-Lipo pulmonary mucosal delivery promotes lung residency and draining lymph node activation

**Figure 7:**
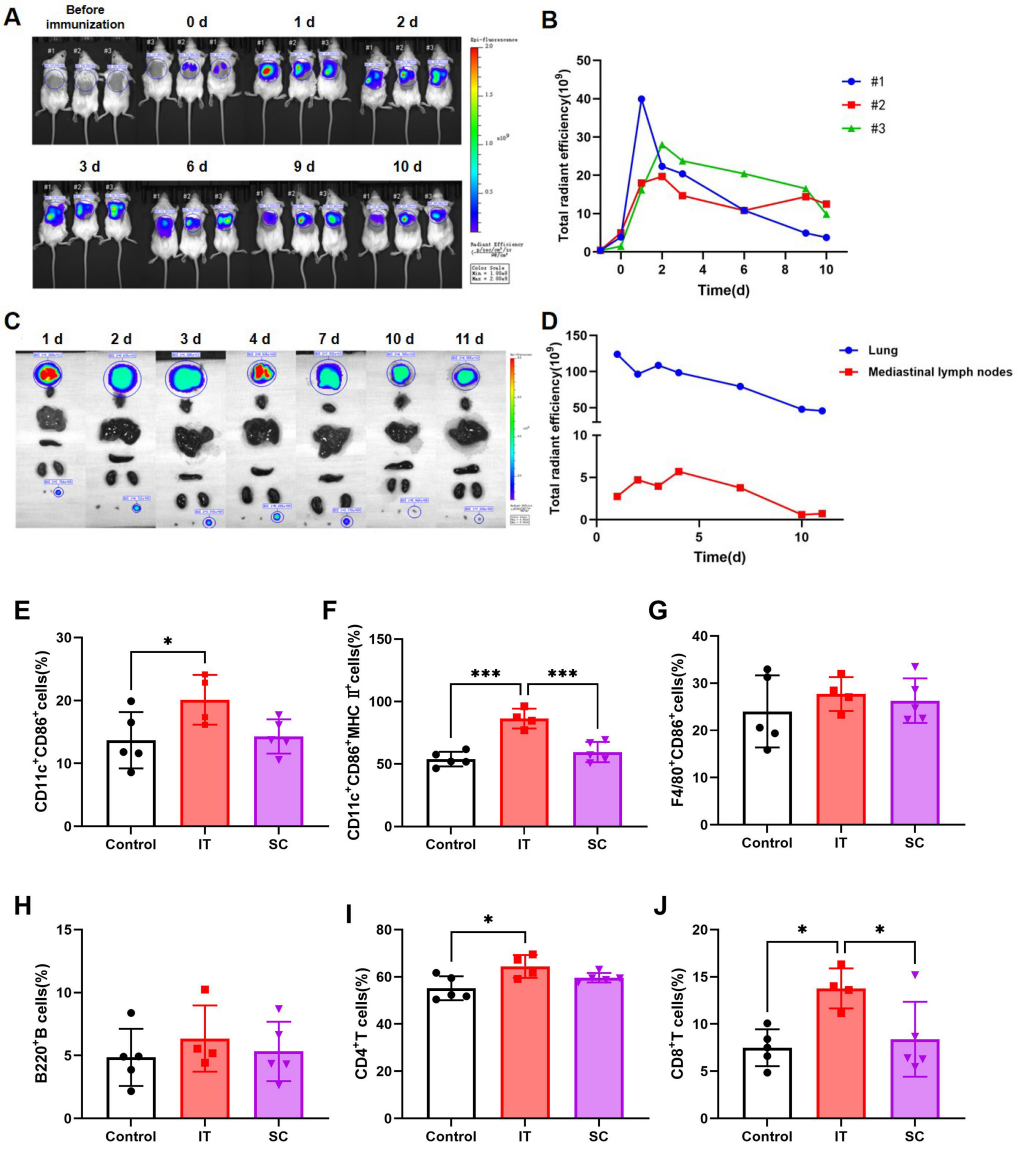
*In vivo* biodistribution and immune-cell activation associated with pulmonary mucosal administration of S_450-471_@DDA-Lipo. **(A)** *In vivo* fluorescence imaging of the lung region; **(B)** quantitative analysis of fluorescence intensity *in vivo*; **(C)** ex vivo imaging of isolated organs and lymph nodes; **(D)** quantitative analysis of ex vivo fluorescence intensity. The isolated organs shown from top to bottom and left to right are lung, heart, liver, spleen, kidney, cervical lymph nodes, axillary lymph nodes, and mediastinal lymph nodes. **(E)** proportion of CD11c+CD86+ dendritic cells in the lung; **(F)** proportion of CD11c+CD86+MHC II+ dendritic cells in the lung; **(G)** proportion of F4/80+CD86+ cells in the lung; **(H)** proportion of B cells in mediastinal lymph nodes; **(I)** proportion of CD4+ T cells in mediastinal lymph nodes; **(J)** proportion of CD8+ T cells in mediastinal lymph nodes. N = 3 per group for **(A–D)** and N = 5 per group for **(E–J)**. Statistical analysis was performed by one-way ANOVA followed by Tukey’s multiple comparisons test unless otherwise indicated. *P < 0.05; **P < 0.01; ***P < 0.001; ****P < 0.0001.

To elucidate the mechanisms underlying the immunogenicity of S_450-471_@DDA-Lipo, we investigated its biodistribution via the pulmonary mucosal pathway and its subsequent activation of innate and adaptive immune cells in the relevant tissues. After immunization with DDA-Lipo though the pulmonary mucosal route, *in vivo* fluorescence imaging revealed that the formulation was effectively deposited and retained in the lungs for an extended period (up to 10 days) following pulmonary administration, and the distribution pattern gradually became uniform (**Figures 7A, B**). Ex vivo imaging of anatomical organs confirmed that S_450-471_@DDA-Lipo is primarily localized in the lungs and, importantly, identified the mediastinal lymph nodes (MLNs) as the primary draining lymphatic sites where formulation signals accumulate over time (**Figures 7C, D**). This precise targeting of lung tissue and its associated draining lymph nodes provided the anatomical basis for localized immune activation.

We then investigated whether this continuous formulation presence in the lungs led to the activation of local antigen-presenting cells (**Figures 7E–G**), which are critical for initiating the immune response. In this study, CD11c^+^CD86^+^cells were used to represent activated dendritic cells, whereas CD11c^+^CD86^+^MHC II^+^ cells were considered a more stringently defined activated antigen-presenting dendritic-cell subset. Compared with the control group, the IT group showed a significant increase in the proportion of CD11c^+^CD86^+^DCs (P < 0.05) and CD11c^+^CD86^+^MHC II^+^DCs (P < 0.001) in the lung single-cell suspension, while the SC group showed significant difference. No statistically significant difference was observed in the percentage of F4/80^+^CD86^+^ cells between the IT group and the SC group. The significant activation of lung DCs, specialized in migrating to draining lymph nodes and priming T cells, suggested that antigenic signals were effectively relayed to the MLNs. Consistent with this, analysis of vaccine-drained MLNs showed adaptive immune priming (**Figures 7H–J**). Compared with the control group, IT significantly enhanced the proliferation of CD4^+^T cells (P < 0.05) and CD8^+^T cells (P < 0.05). In contrast, the SC group did not provoke a significant proliferative response.

## 4 Discussion

In the present study, we established and preliminarily evaluated a DDA cationic liposome-based pulmonary mucosal immunization platform using a SARS-CoV-2 spike-derived branched peptide as a model antigen. Rather than a definitive efficacy study, the current work should be interpreted as a proof-of-concept investigation of route feasibility, initial immunogenic potential, and selected mechanistic correlates. The main contribution of this study is the preliminary construction and evaluation of a DDA-liposome-based pulmonary mucosal peptide formulation in this experimental setting.

In this study, DDA cationic liposomes were prepared by film-dispersion method. The resulting nanoparticles showed a mean particle size of approximately 101.6 ± 4.16 nm, a PDI of 0.271 ± 0.032, and a zeta potential of +42.67 ± 3.50 mV. Previous studies have shown that smaller particles generally exhibit better mucus permeability, which facilitates penetration through the mucus barrier and enhances contact with antigen-presenting cells in the mucosal epithelium as well as access to draining lymph nodes (28, 29). Therefore, maintaining the particle size below 200 nm is considered important for efficient initiation of mucosal immune responses (30). In addition, the strong positive surface charge of DDA-based liposomes may promote electrostatic interaction with negatively charged mucosal surfaces and dendritic cells, and this property may be associated with prolonged pulmonary retention and enhanced cellular uptake, as suggested by previous reports (20, 31). However, because charge-variant control liposomes were not included in the present study, the specific contribution of surface charge to these effects was not directly established here.

Our *in vitro* data further supported the immune-promoting potential of the formulation. Previous studies have shown that positively charged cationic liposomes can enhance the interaction between liposomes and dendritic cells, thereby promoting antigen uptake and increasing the expression of activation-associated molecules such as MHC II, CD80, CD86, and CD40 (32, 33). S_450-471_@DDA-Lipo+CpG in this study can also significantly increase the expression of MHC II, CD80 and CD86 on DCs, indicating that S_450-471_@DDA-Lipo+CpG can effectively promote the activation and maturation of DCs, and mature DCs migrate from peripheral tissues to lymph nodes, where they present antigens to T lymphocytes and trigger adaptive immune responses (34). In addition, S_450-471_@DDA-Lipo+CpG also promoted the secretion of IFN-γ by DCs, which is an important Th1 cytokine, which can promote DCs to secrete more IL-1β and IL-12p70, further effectively activate CD4^+^T cells and CD8^+^T cells (35, 36). In addition, the present *in vitro* characterization was performed under a single tested formulation condition. Although no detectable cytotoxicity was observed in BMDCs at this concentration, we did not perform dose-dependent toxicity or uptake optimization studies. Moreover, direct visualization of liposome uptake by dendritic cells and intracellular localization analyses were not included in the present work. Therefore, the mechanistic interpretation of cellular uptake and intracellular trafficking remains preliminary and should be further investigated in future studies.

The *in vivo* results showed that the formulation induced high levels of antigen-specific IgG in serum and antigen-specific IgA in BALF after pulmonary mucosal immunization, suggesting that the formulation not only stimulated systemic humoral immunity, but also effectively activated local mucosal immunity. The apparent difference between antigen-specific IgA titer and total IgA concentration in BALF is not contradictory, because these two measurements reflect different aspects of the mucosal humoral response. Specifically, pulmonary immunization preferentially increased peptide-specific IgA responses, whereas the total mucosal IgA pool changed only modestly. It is worth noting that pulmonary mucosal immunization shows better immune effect than nasal mucosal immunization, which may be related to the longer retention time of the formulation in the alveolar area and wider antigen distribution (37). suggesting that pulmonary mucosal immunization is more suitable for this formulation.

At the same time, the translational relevance of the present intratracheal model should be interpreted cautiously. Orotracheal intratracheal administration in mice was used here as a controlled experimental approach to ensure reproducible deposition of the formulation into the lower respiratory tract, rather than as a proposed final clinical vaccination procedure. The long-term objective of this work is to support the future development of an inhalable or nebulized pulmonary delivery system. Therefore, the present findings should be interpreted as proof-of-concept evidence for lung-directed mucosal immunization, while future translation will require adaptation of the formulation to clinically practical aerosolized or nebulized delivery platforms.

The serum IgG subtype profile further suggested mixed Th1/Th2-oriented systemic immune activation. IgG2a is generally associated with Th1-type responses and IFN-γ-related immune polarization, whereas IgG1 is more commonly associated with Th2-type responses and IL-4-related immunity (38). In our study, antigen-specific IgG1 and IgG2a titers increased after immunization, together with increased serum IFN-γ and IL-4 levels, supporting the induction of a mixed Th1/Th2 immune profile. Notably, because IgG1 levels were higher than IgG2a, the humoral response should not be interpreted as purely Th1-polarized, but rather as reflecting a mixed Th1/Th2 profile with a substantial Th2-associated component. The increase in serum Granzyme B further suggested enhanced cytotoxic immune-associated activity after immunization, although direct functional assessment of NK-cell or CTL killing activity was not performed (39). IL-10 levels did not change significantly, suggesting that overt systemic immunosuppressive effects were not observed under the tested conditions (40).

The splenic memory T-cell data, however, should be interpreted cautiously. After ex vivo restimulation with the S_450–471_ peptide, no statistically significant differences were observed among the groups in splenic central memory or effector memory T-cell subsets. Therefore, no definitive conclusion can be drawn regarding route-dependent effects on splenic memory T-cell differentiation under the current experimental conditions. In addition, because an empty-liposome-only control group was not included in the *in vivo* study, the present data do not allow us to determine whether any nonsignificant numerical variation observed in splenic T-cell memory subsets may be attributable to the liposomal carrier itself. Future studies including an empty-liposome control group will be required to clarify the contribution of the carrier to systemic memory T-cell phenotypes.

In contrast to the relatively limited splenic memory findings, pulmonary mucosal immunization was associated with much more pronounced local immune changes in the lung. Flow cytometric analysis performed after ex vivo restimulation with the S450–471 peptide revealed marked changes in pulmonary immune-cell phenotypes, particularly in the IT and SC+IT groups. Pulmonary mucosal immunization significantly increased the proportions of activated B cells and T cells in the lung and promoted the formation of CD4^+^ and CD8^+^TRM-like phenotypes. These findings support the ability of the formulation to enhanced peptide-restimulated local immune phenotypes in the respiratory mucosa. However, these data should be interpreted with appropriate caution. Another limitation is that peptide-alone, CpG-alone, empty-liposome, and empty-liposome-plus-CpG control groups were not included in the *in vivo* immunization experiments. Therefore, the relative contribution of the antigen, carrier, and adjuvant to the observed immune readouts cannot be fully distinguished at present. In addition, intravascular antibody labeling or vascular perfusion was not performed before tissue processing. Although the markers used to define TRM-like cells in this study are not expected to be expressed on naïve or central memory T cells circulating in the blood of healthy mice, more rigorous *in vivo* approaches will still be required in future studies to precisely quantify the true tissue-resident population.

The local mechanistic observations are also consistent with the idea that pulmonary retention and regional lymphoid exposure contribute to mucosal immune activation. Bronchial associated lymphoid tissue (BALT) is a mucosal lymphoid follicle composed of B cell and T cell regions in the respiratory tract, which is the site of the initial immune response to inhaled antigens. According to previous studies, inhaled antigens may be sampled by M cells and dendritic cells in the BALT-associated epithelium, followed by antigen presentation and downstream activation of adaptive immune responses. Subsequently, DCs present antigens through MHC, which in turn activate CD4^+^ and CD8^+^ T cells. This process of T cell activation is a key step in driving the proliferation of B cells and ultimately their differentiation into IgA-secreting plasma cells (6–8). In the present study, *in vivo* imaging showed that S_450-471_@DDA-Lipo was retained in the lungs for at least 10 days, which may favor sustained local antigen exposure. In addition, the fluorescent formulation was detected in mediastinal lymph nodes, suggesting that the formulation reached the draining lymph-node region and was associated with local immune activation. Together with the increased proportions of activated dendritic-cell phenotypes in the lung and the increased lymphocyte proportions in mediastinal lymph nodes, these findings support the view that pulmonary delivery of the formulation facilitates a local mucosal immune environment linked to downstream adaptive immune activation.

In summary, this study provides a proof-of-concept pulmonary mucosal immunization platform based on DDA cationic liposomes for inducing mucosal and systemic immune responses against a SARS-CoV-2 spike-derived peptide antigen. The findings support the immunogenicity and mechanistic potential of this platform, while the interpretation of protective efficacy should remain cautious. Several limitations should be acknowledged. Neutralizing antibody activity and *in vivo* protective efficacy were not evaluated in the present study. *In vitro* antigen release kinetics were not assessed and should be investigated in future work to better define formulation stability and release behavior. Some numerical differences observed in systemic immune-cell subsets did not reach statistical significance, and larger sample sizes will be needed to determine whether these reflect true biological effects. In addition, longitudinal antibody kinetics were evaluated only in the IT group, so direct comparison of response durability among different administration routes was not possible. Histopathological examination of lung tissue was not performed, and cell-type-specific uptake analyses *in vivo* were not conducted. Future studies incorporating neutralization assays, viral challenge experiments, antigen-release analysis, histological evaluation, controlled peptide-restimulation assays, and cell-resolved uptake analyses will further strengthen the evaluation of this immunization platform.

## Data Availability

The raw data supporting the conclusions of this article will be made available by the authors, without undue reservation.
