# Supplementary material for: Preliminary evaluation of a DDA cationic liposome-based pulmonary mucosal immunization platform carrying a SARS-CoV-2 spike-derived branched peptide
Source: Front Immunol. 2026 May 29;17:1824741. doi: 10.3389/fimmu.2026.1824741 (PMC13260544; doi:10.3389/fimmu.2026.1824741)
Supplement: Supplementary file 1 [file DataSheet1.pdf]

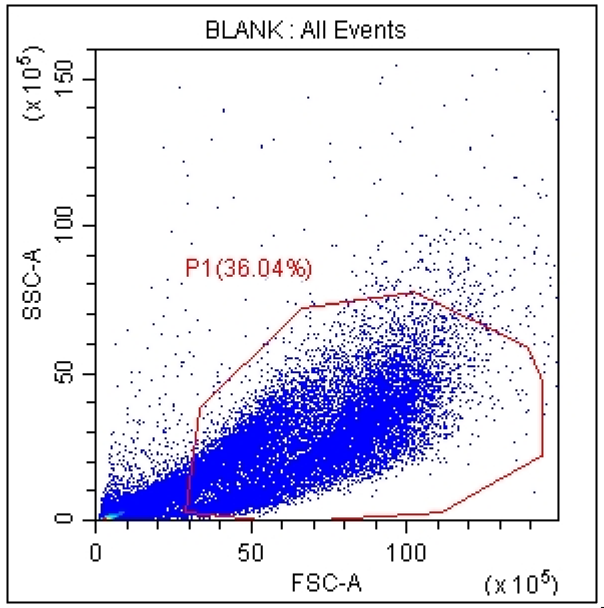

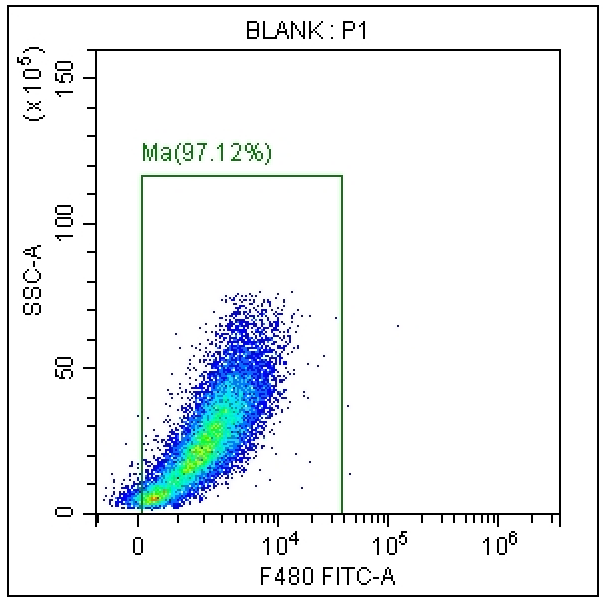

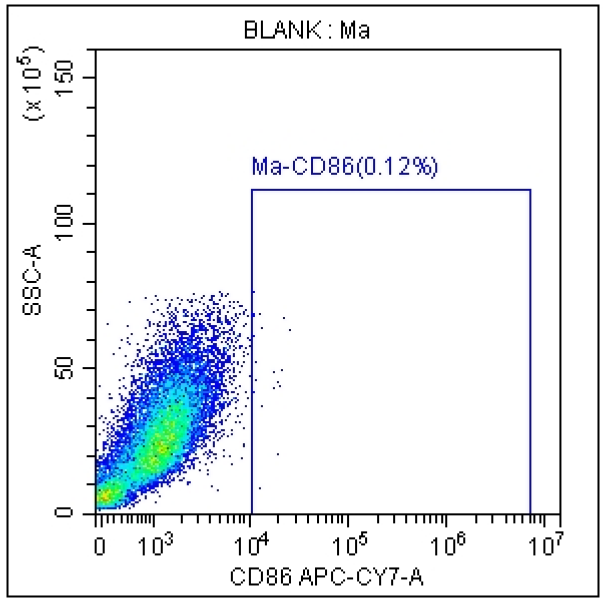

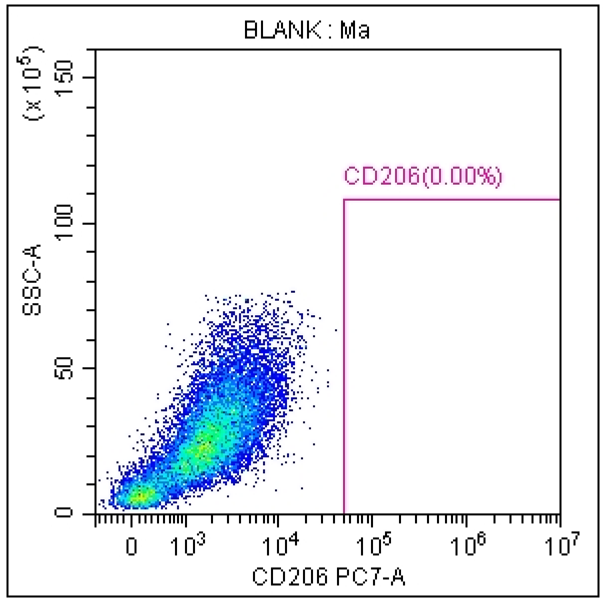

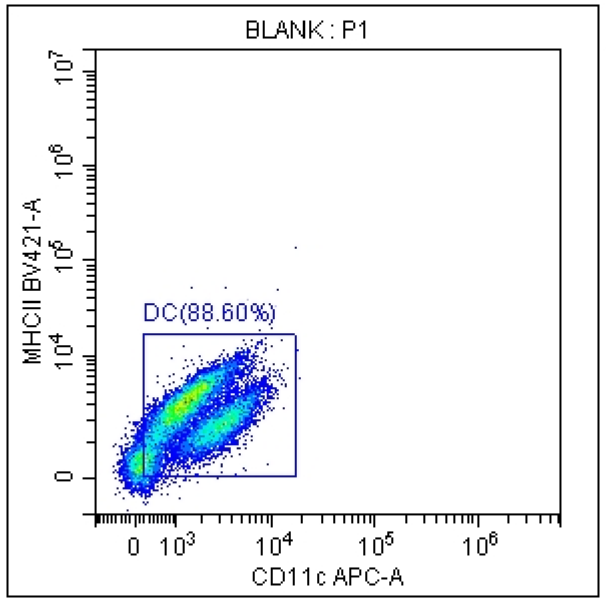

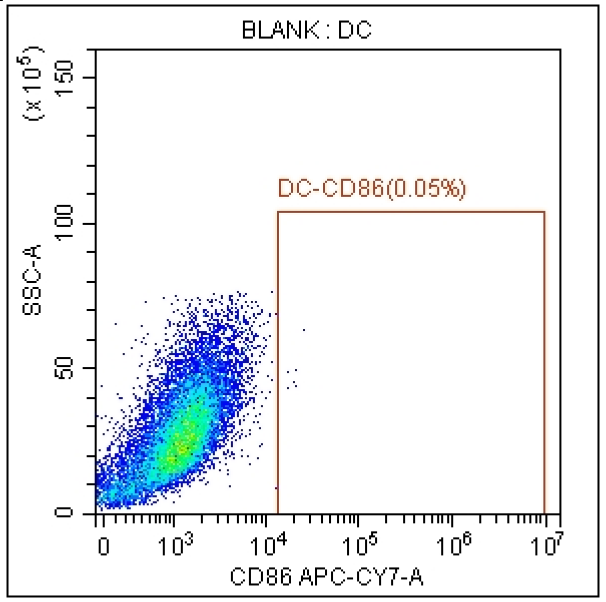

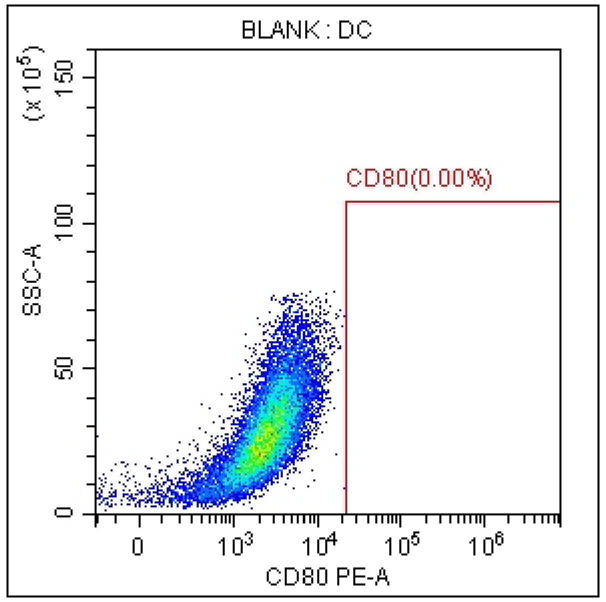

试管名称： BLANK

样本ID：

群体	颗粒数	%父群	%总数
All Events	47117	100.00%	100.00%
P1	16980	36.04%	36.04%
Ma	16491	97.12%	35.00%
DC	15044	88.60%	31.93%
Ma-CD86	19	0.12%	0.04%
CD206	0	0.00%	0.00%
DC-CD86	8	0.05%	0.02%
CD80	0	0.00%	0.00%
CD11c	4	0.02%	0.01%
P2	1	25.00%	0.00%
P3	1	25.00%	0.00%
P4	3	75.00%	0.01%

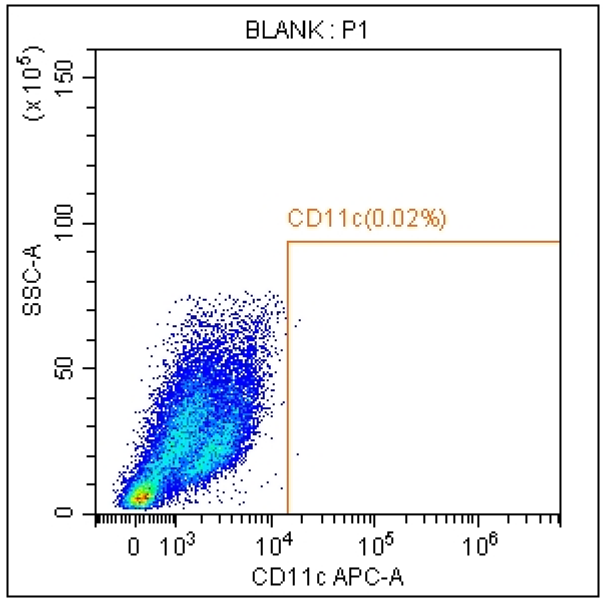

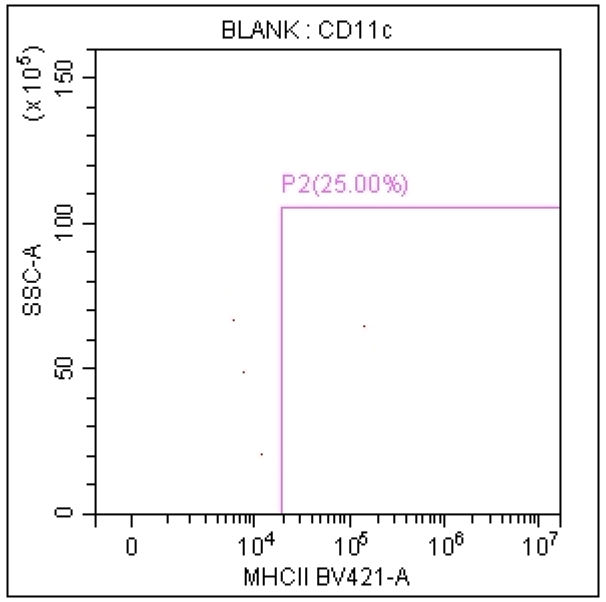

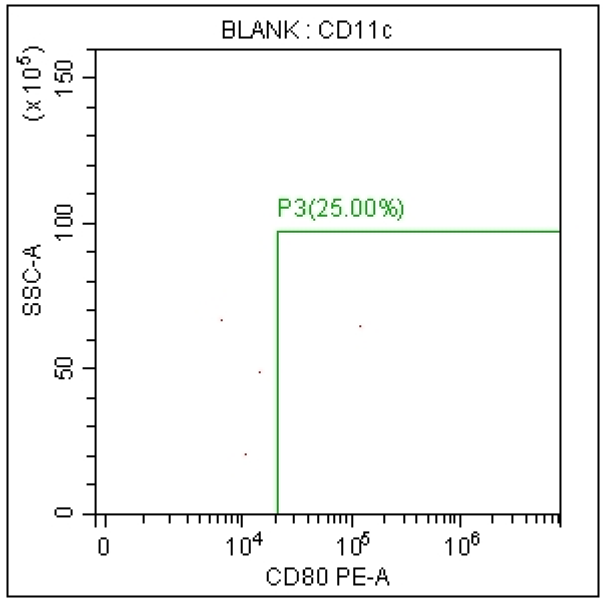

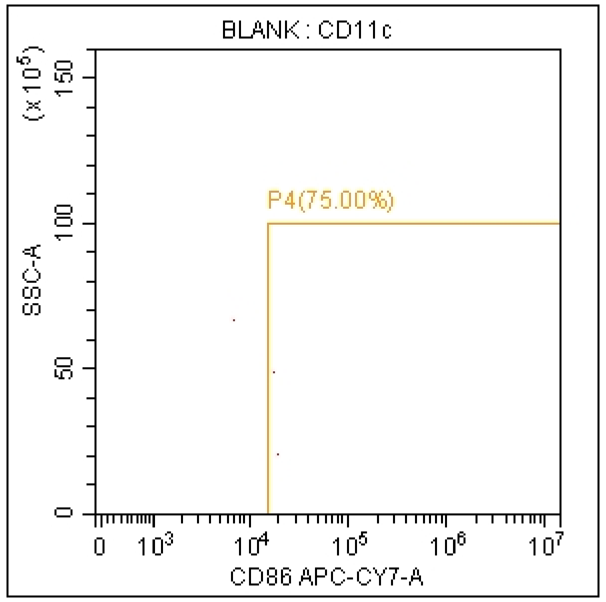

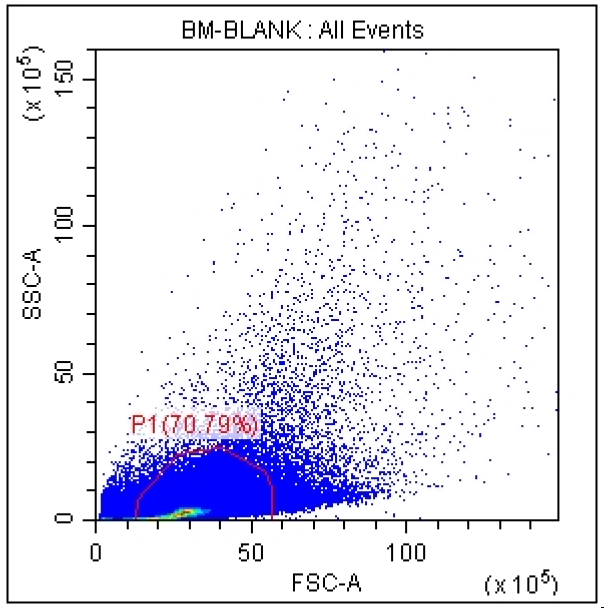

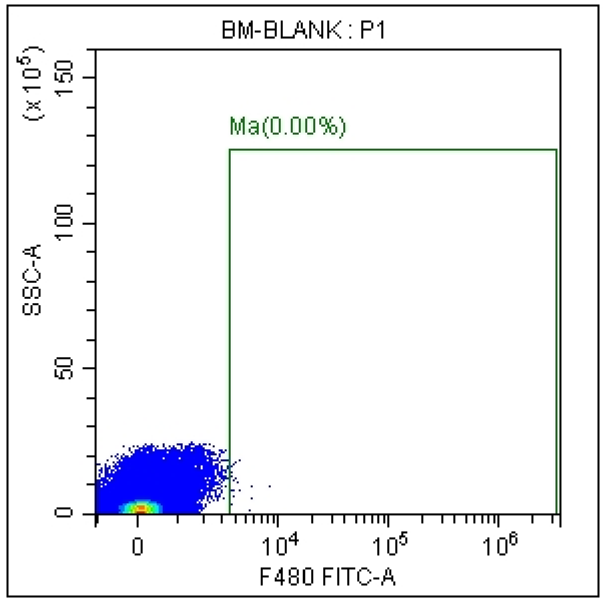

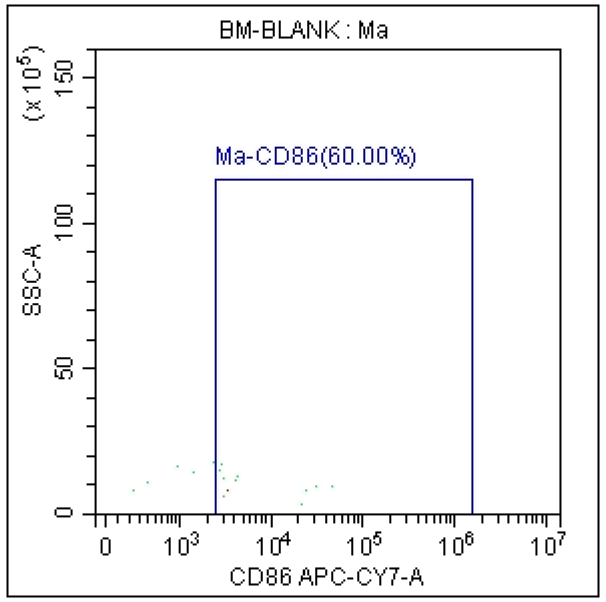

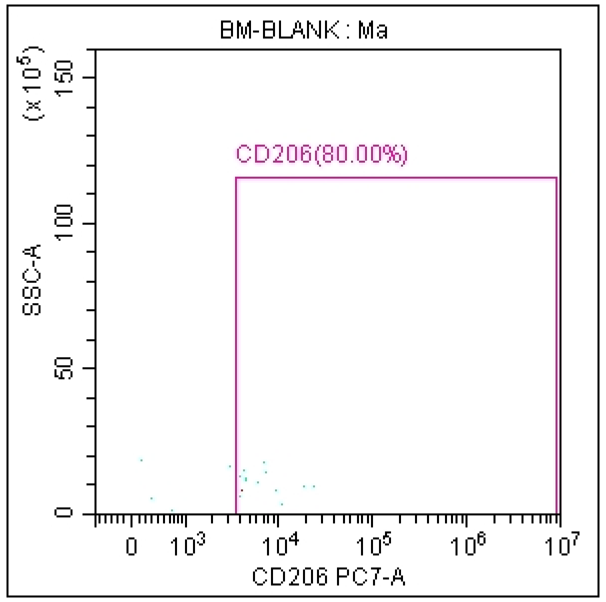

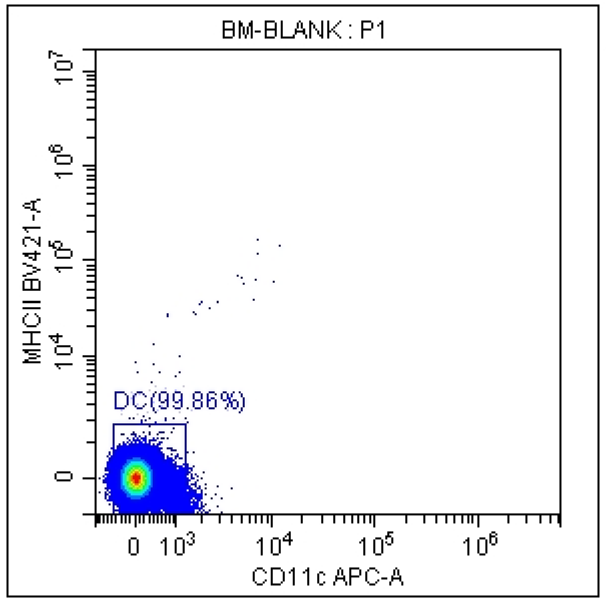

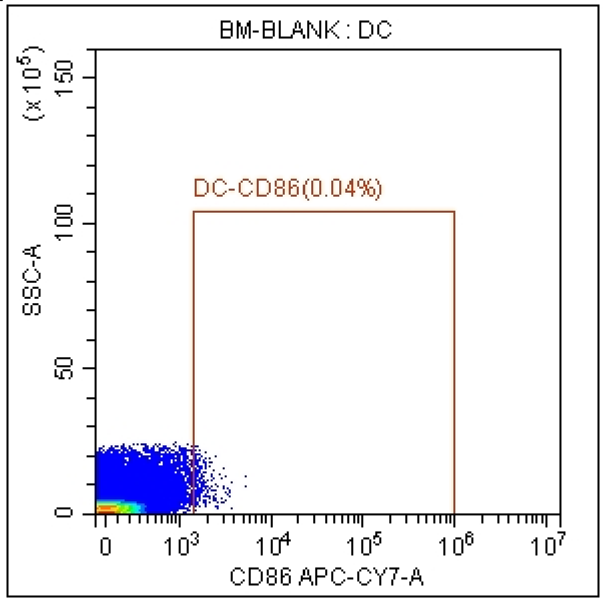

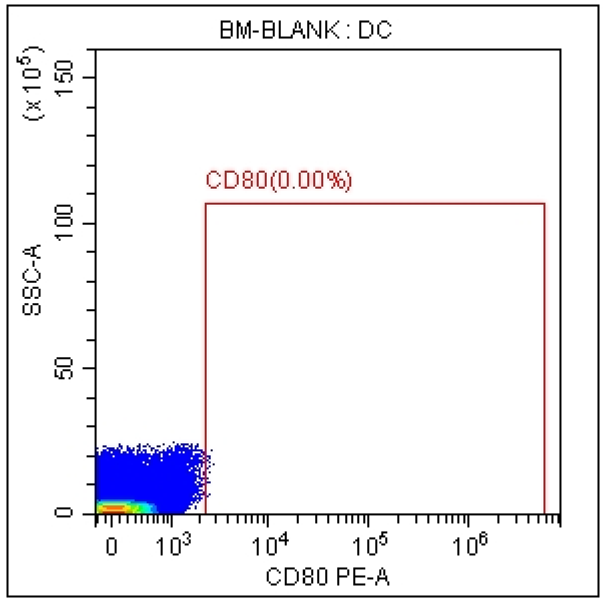

试管名称： BM-BLANK

样本ID：

群体	颗粒数	%父群	%总数
All Events	954536	100.00%	100.00%
P1	675718	70.79%	70.79%
Ma	20	0.00%	0.00%
DC	674797	99.86%	70.69%
Ma-CD86	12	60.00%	0.00%
CD206	16	80.00%	0.00%
DC-CD86	270	0.04%	0.03%
CD80	19	0.00%	0.00%
CD11c	0	0.00%	0.00%
P2	0	####	0.00%
P3	0	####	0.00%
P4	0	####	0.00%

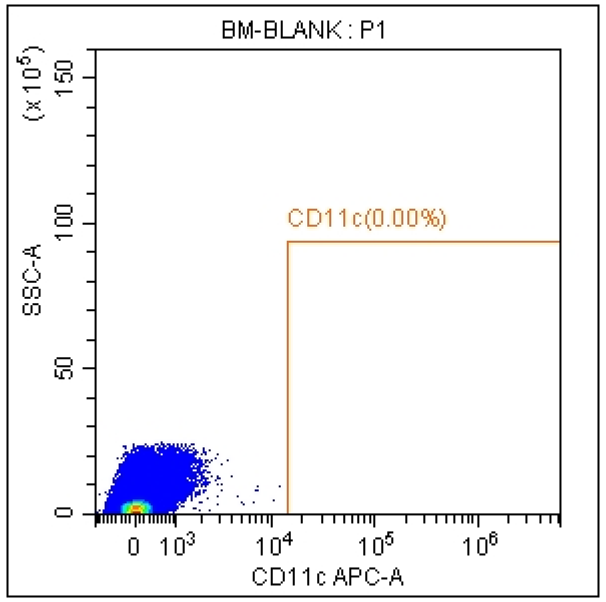

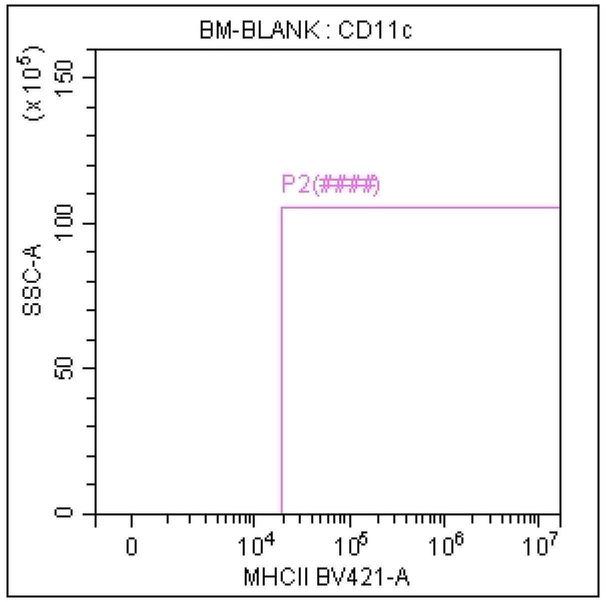

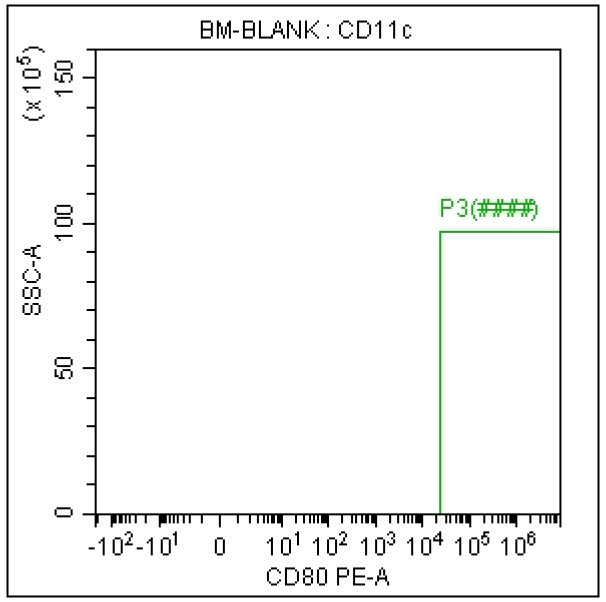

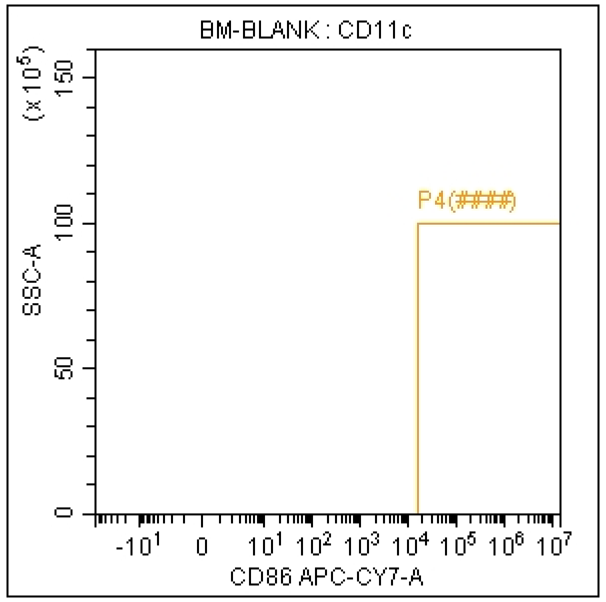

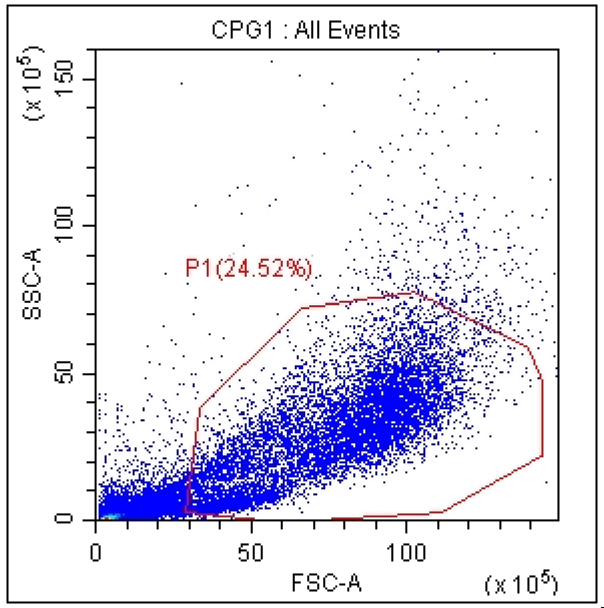

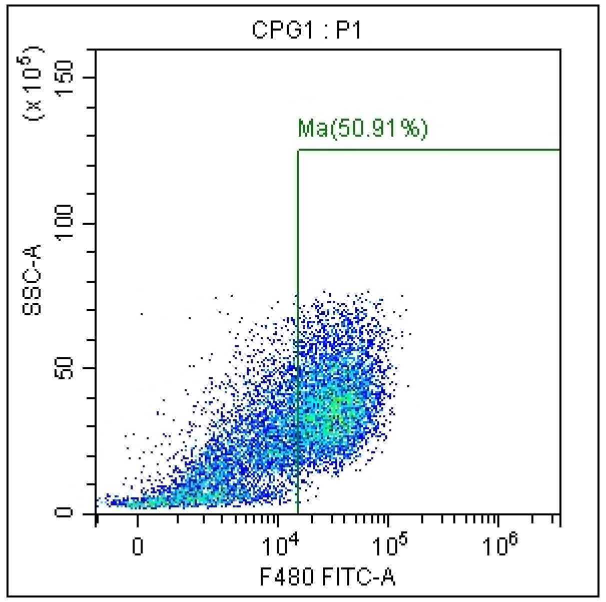

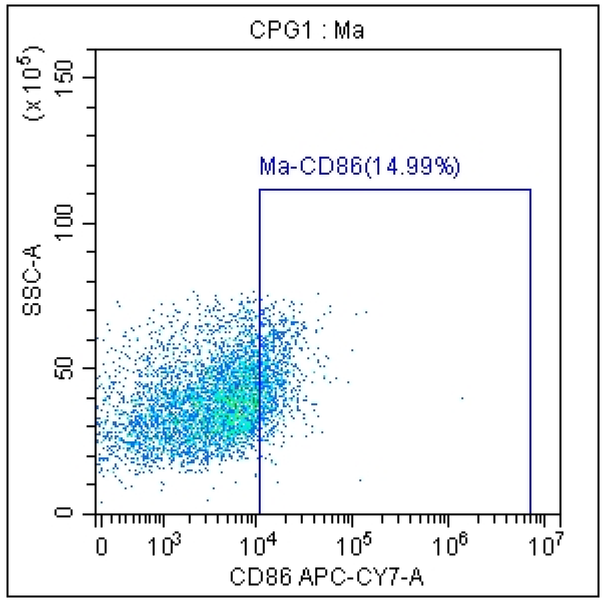

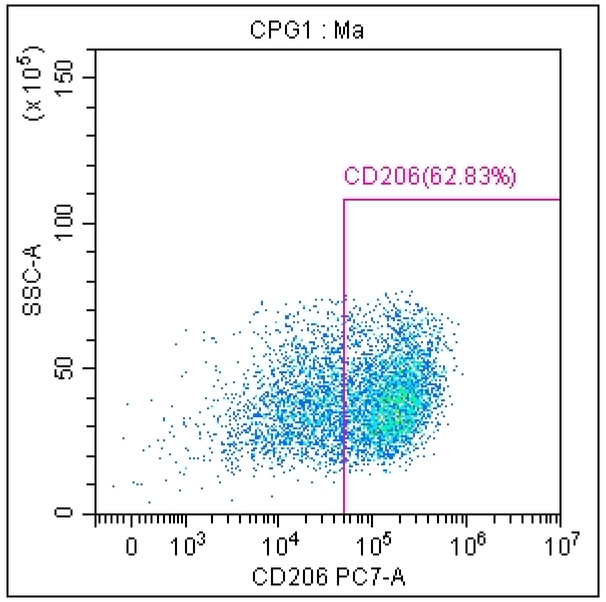

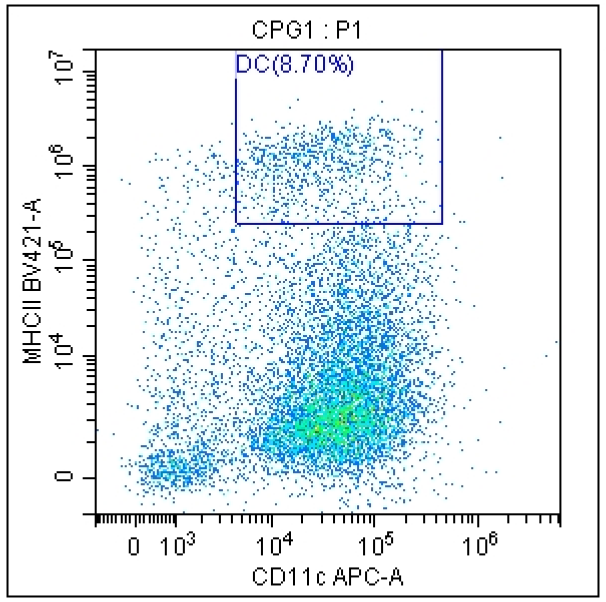

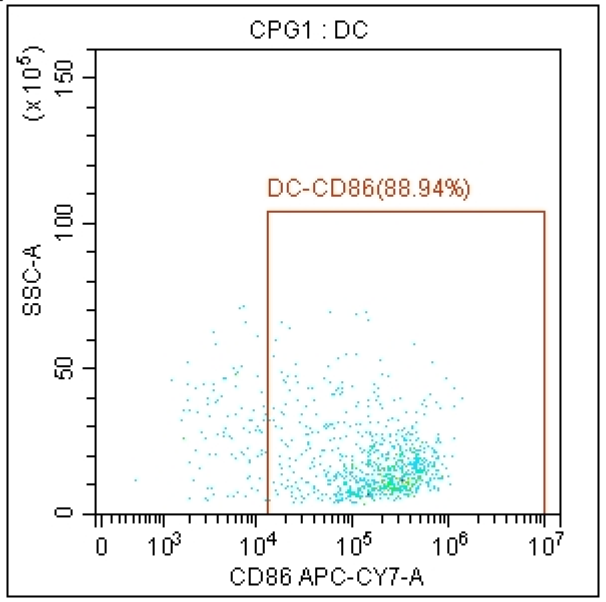

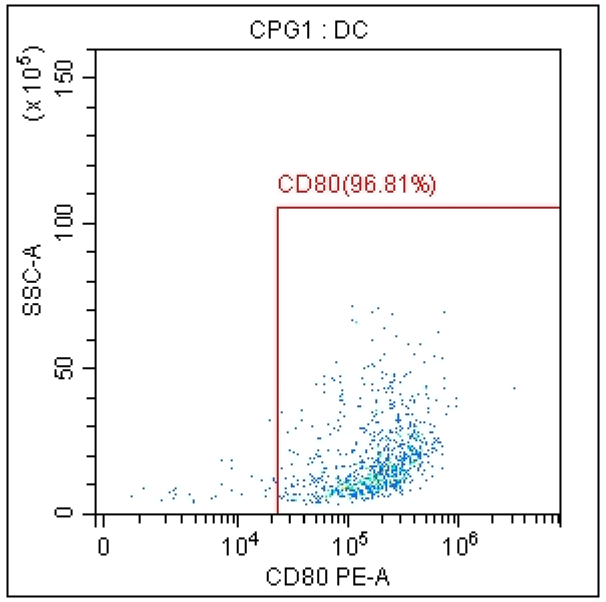

试管名称： CPG1
样本ID：

群体	颗粒数	%父群	%总数
All Events	41103	100.00%	100.00%
P1	10079	24.52%	24.52%
Ma	5131	50.91%	12.48%
DC	877	8.70%	2.13%
Ma-CD86	769	14.99%	1.87%
CD206	3224	62.83%	7.84%
DC-CD86	780	88.94%	1.90%
CD80	849	96.81%	2.07%
CD11c	7558	74.99%	18.39%
P2	1830	24.21%	4.45%
P3	6433	85.12%	15.65%
P4	1094	14.47%	2.66%

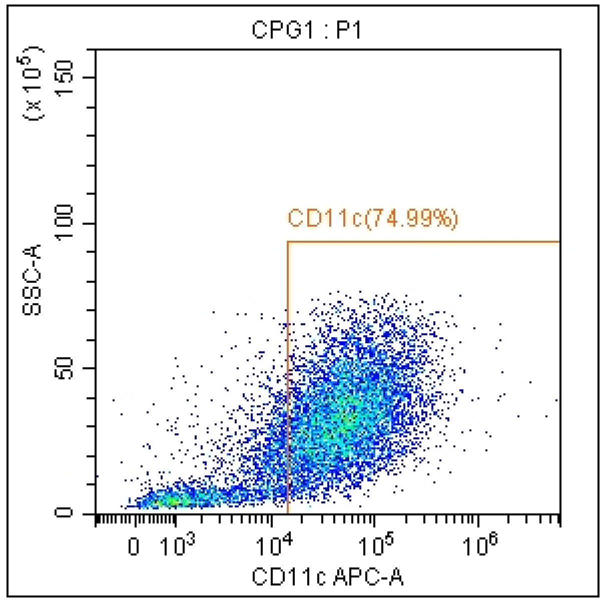

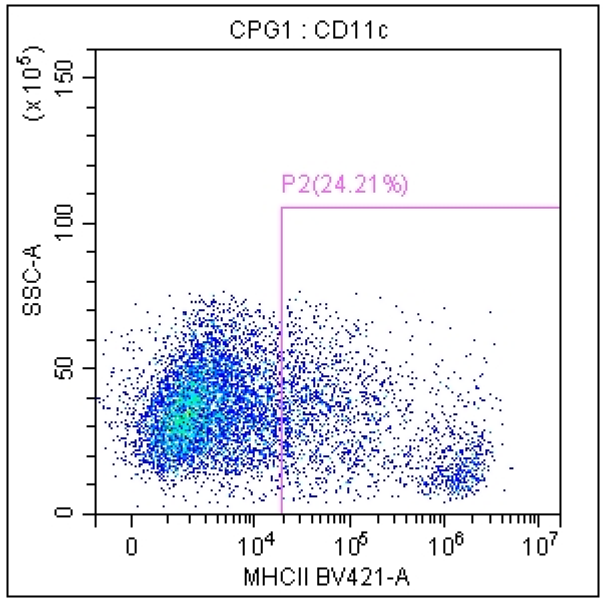

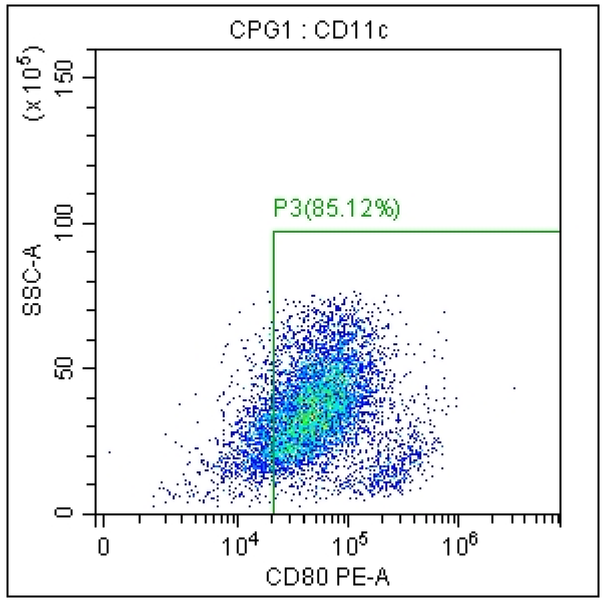

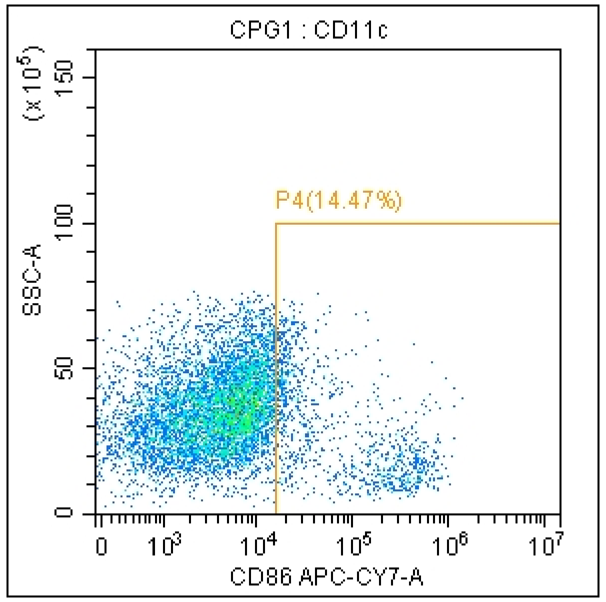

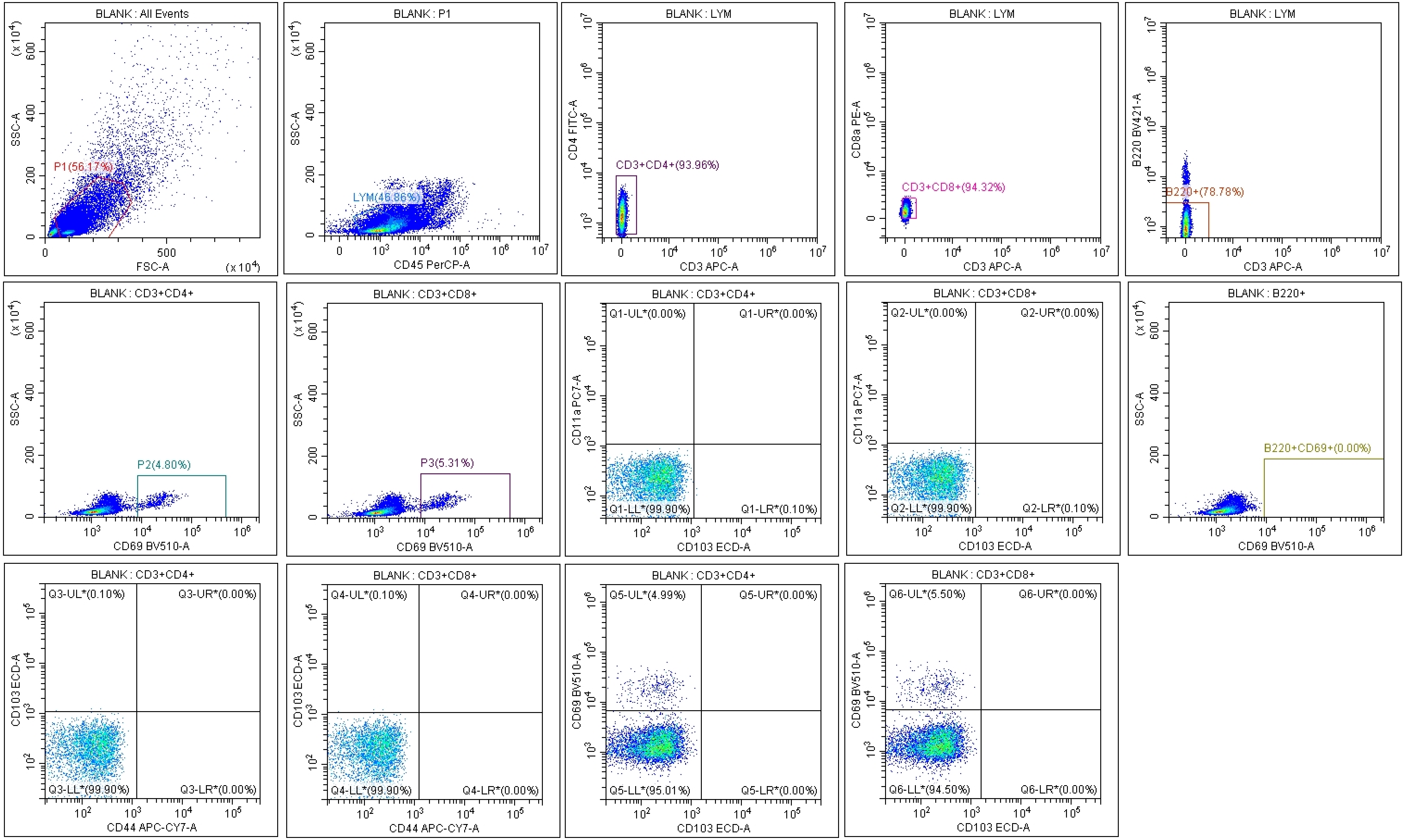
BLANK : All Events
P1(56.17%)
SSC-A
FSC-A
BLANK : P1
LYM(46.86%)
SSC-A
CD45 PerCP-A
BLANK : LYM
CD3+CD4+(93.96%)
CD4 FITC-A
CD3 APC-A
BLANK : LYM
CD3+CD8+(94.32%)
CD8a PE-A
CD3 APC-A
BLANK : LYM
B220+(78.78%)
B220 BV421-A
CD3 APC-A
BLANK : CD3+CD4+
P2(4.80%)
SSC-A
CD69 BV510-A
BLANK : CD3+CD8+
P3(5.31%)
SSC-A
CD69 BV510-A
BLANK : CD3+CD4+
Q1-UL*(0.00%)
Q1-UR*(0.00%)
Q1-LL*(99.90%)
Q1-LR*(0.10%)
CD11a PC7-A
CD103 ECD-A
BLANK : CD3+CD8+
Q2-UL*(0.00%)
Q2-UR*(0.00%)
Q2-LL*(99.90%)
Q2-LR*(0.10%)
CD11a PC7-A
CD103 ECD-A
BLANK : B220+
B220+CD69+(0.00%)
SSC-A
CD69 BV510-A
BLANK : CD3+CD4+
Q3-UL*(0.10%)
Q3-UR*(0.00%)
Q3-LL*(99.90%)
Q3-LR*(0.00%)
CD103 ECD-A
CD44 APC-CY7-A
BLANK : CD3+CD8+
Q4-UL*(0.10%)
Q4-UR*(0.00%)
Q4-LL*(99.90%)
Q4-LR*(0.00%)
CD103 ECD-A
CD44 APC-CY7-A
BLANK : CD3+CD4+
Q5-UL*(4.99%)
Q5-UR*(0.00%)
Q5-LL*(95.01%)
Q5-LR*(0.00%)
CD69 BV510-A
CD103 ECD-A
BLANK : CD3+CD8+
Q6-UL*(5.50%)
Q6-UR*(0.00%)
Q6-LL*(94.50%)
Q6-LR*(0.00%)
CD69 BV510-A
CD103 ECD-A

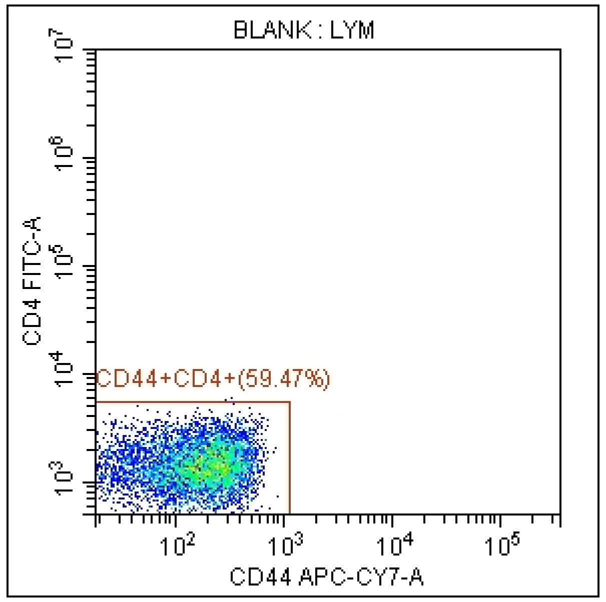

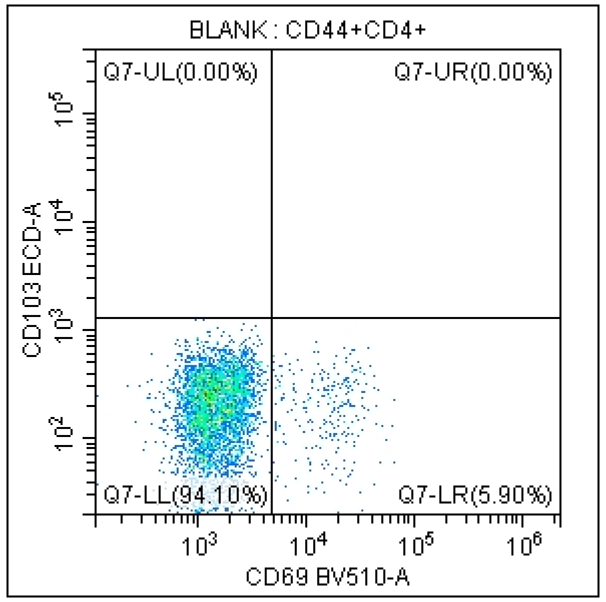

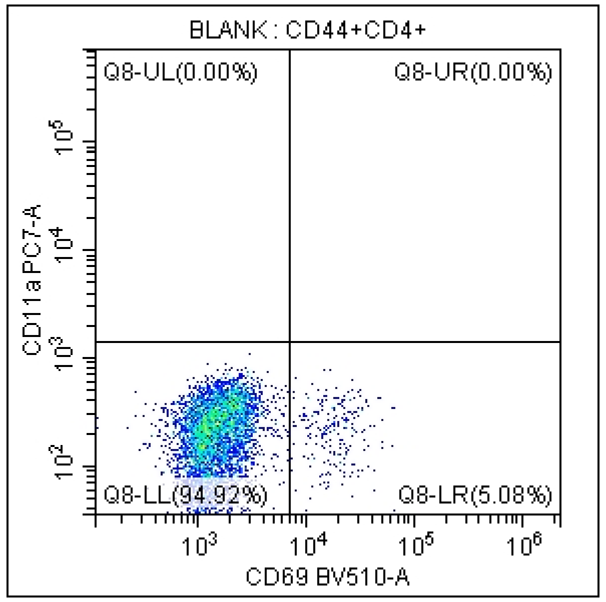

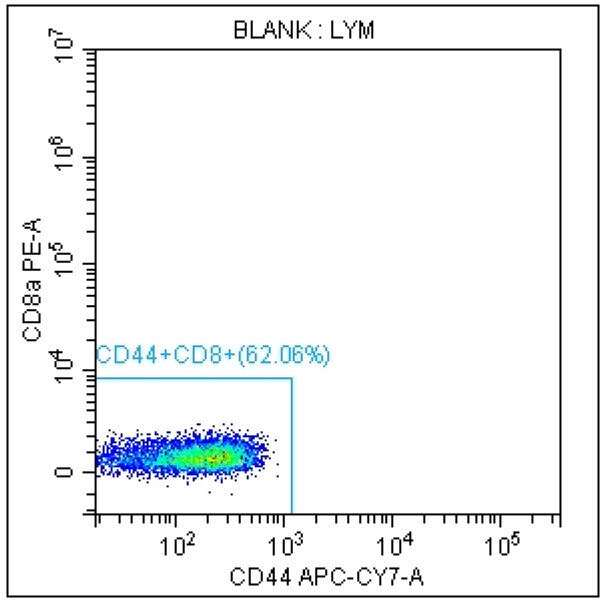

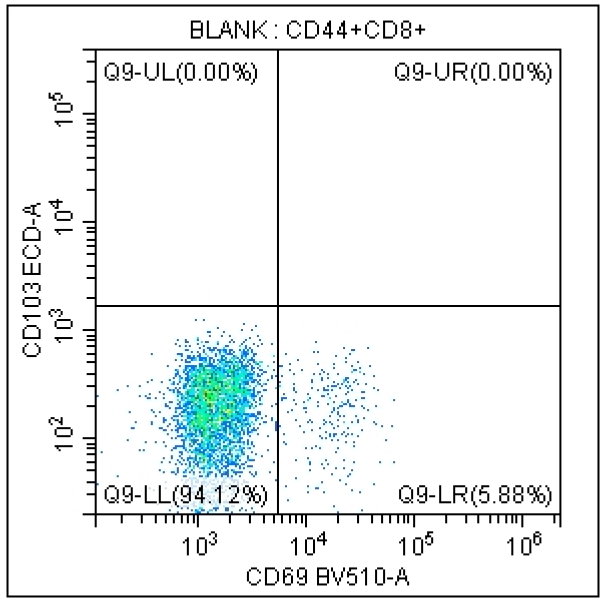

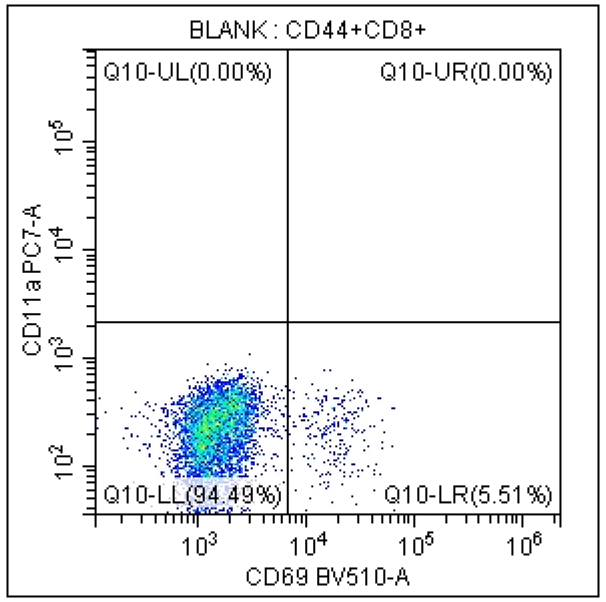

试管名称： BLANK
样本ID：

群体	颗粒数	%父群	%总数
All Events	37988	100.00%	100.00%
P1	21339	56.17%	56.17%
LYM	10000	46.86%	26.32%
CD3+CD4+	9396	93.96%	24.73%
CD3+CD8+	9432	94.32%	24.83%
B220+	7878	78.78%	20.74%
P2	451	4.80%	1.19%
P3	501	5.31%	1.32%
Q1-UR	0	0.00%	0.00%
Q1-UL	0	0.00%	0.00%
Q1-LR	9	0.10%	0.02%
Q2-UR	0	0.00%	0.00%
Q2-UL	0	0.00%	0.00%
Q2-LR	9	0.10%	0.02%
Q3-UR	0	0.00%	0.00%
Q3-UL	9	0.10%	0.02%
Q3-LR	0	0.00%	0.00%
Q4-UR	0	0.00%	0.00%
Q4-UL	9	0.10%	0.02%
Q4-LR	0	0.00%	0.00%
Q5-UR	0	0.00%	0.00%
Q5-UL	469	4.99%	1.23%
Q5-LR	0	0.00%	0.00%
Q6-UR	0	0.00%	0.00%
Q6-UL	519	5.50%	1.37%
Q6-LR	0	0.00%	0.00%
CD44+CD4+	5947	59.47%	15.65%
CD44+CD8+	6206	62.06%	16.34%
B220+CD69+	0	0.00%	0.00%
Q8-UR	0	0.00%	0.00%
Q8-UL	0	0.00%	0.00%
Q8-LR	302	5.08%	0.79%
Q10-UR	0	0.00%	0.00%
Q10-UL	0	0.00%	0.00%
Q10-LR	342	5.51%	0.90%
Q7-UR	0	0.00%	0.00%
Q7-UL	0	0.00%	0.00%
Q7-LL	5596	94.10%	14.73%
Q7-LR	351	5.90%	0.92%
Q9-UR	0	0.00%	0.00%

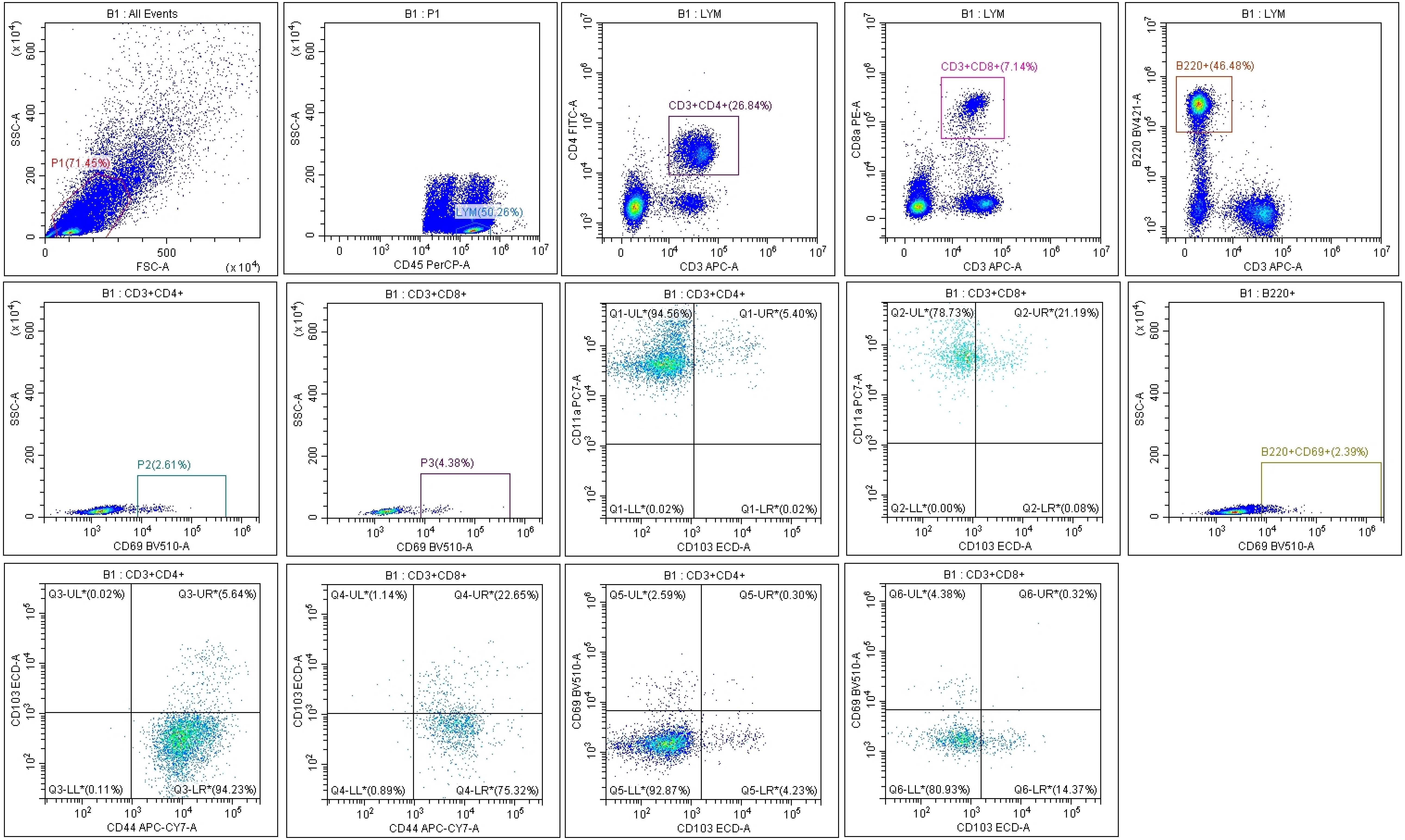

B1 : All Events
P1(71.45%)
SSC-A (x 10^4)
FSC-A (x 10^4)
B1 : P1
LYM(50.26%)
CD45 PerCP-A
B1 : LYM
CD3+CD4+(26.84%)
CD4 FITC-A
CD3 APC-A
B1 : LYM
CD3+CD8+(7.14%)
CD8a PE-A
CD3 APC-A
B1 : LYM
B220+(46.48%)
B220 BV421-A
CD3 APC-A
B1 : CD3+CD4+
P2(2.61%)
CD69 BV510-A
B1 : CD3+CD8+
P3(4.38%)
CD69 BV510-A
B1 : CD3+CD4+
Q1-UL*(94.56%)
Q1-UR*(5.40%)
Q1-LL*(0.02%)
Q1-LR*(0.02%)
CD11a PC7-A
CD103 ECD-A
B1 : CD3+CD8+
Q2-UL*(78.73%)
Q2-UR*(21.19%)
Q2-LL*(0.00%)
Q2-LR*(0.08%)
CD11a PC7-A
CD103 ECD-A
B1 : B220+
B220+CD69+(2.39%)
CD69 BV510-A
B1 : CD3+CD4+
Q3-UL*(0.02%)
Q3-UR*(5.64%)
Q3-LL*(0.11%)
Q3-LR*(94.23%)
CD103 ECD-A
CD44 APC-CY7-A
B1 : CD3+CD8+
Q4-UL*(1.14%)
Q4-UR*(22.65%)
Q4-LL*(0.89%)
Q4-LR*(75.32%)
CD103 ECD-A
CD44 APC-CY7-A
B1 : CD3+CD4+
Q5-UL*(2.59%)
Q5-UR*(0.30%)
Q5-LL*(92.87%)
Q5-LR*(4.23%)
CD69 BV510-A
CD103 ECD-A
B1 : CD3+CD8+
Q6-UL*(4.38%)
Q6-UR*(0.32%)
Q6-LL*(80.93%)
Q6-LR*(14.37%)
CD69 BV510-A
CD103 ECD-A

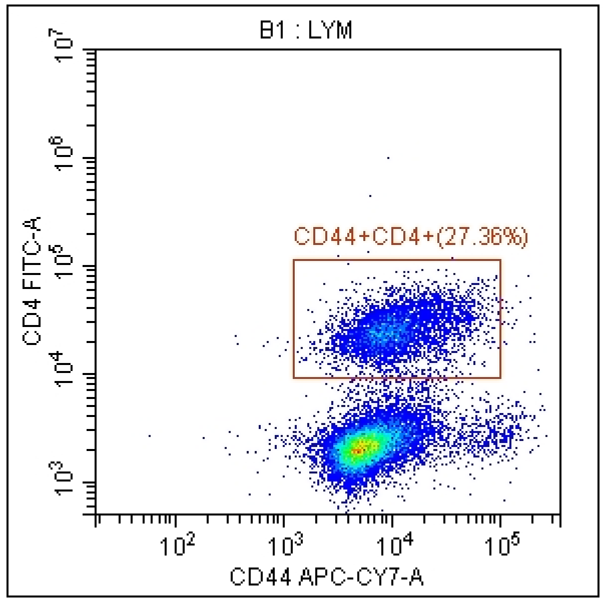

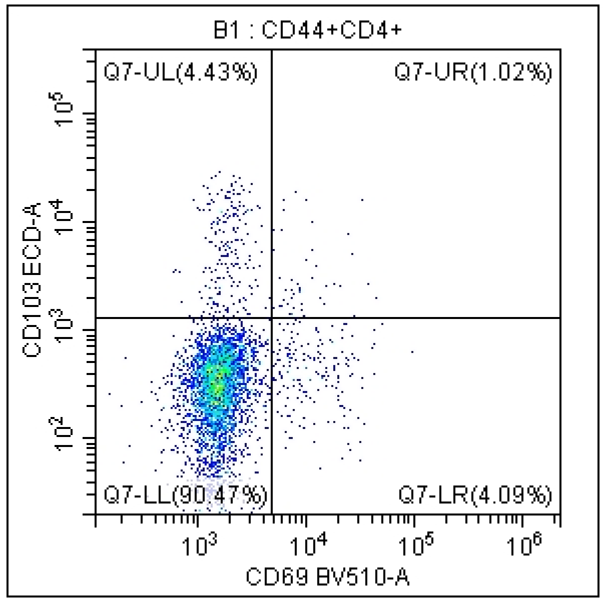

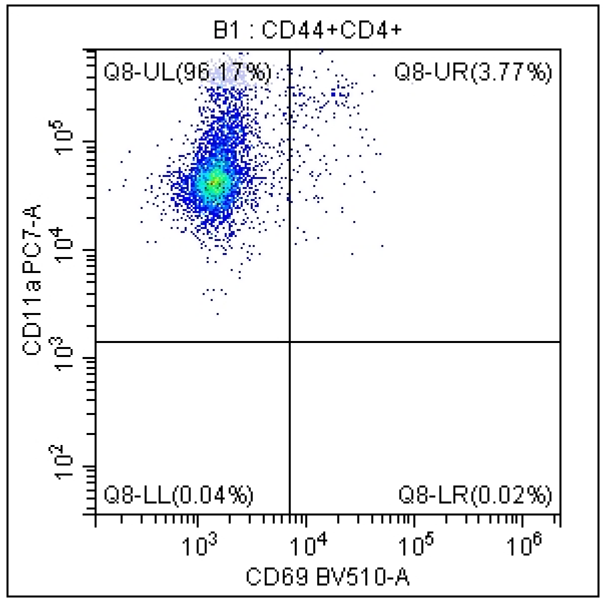

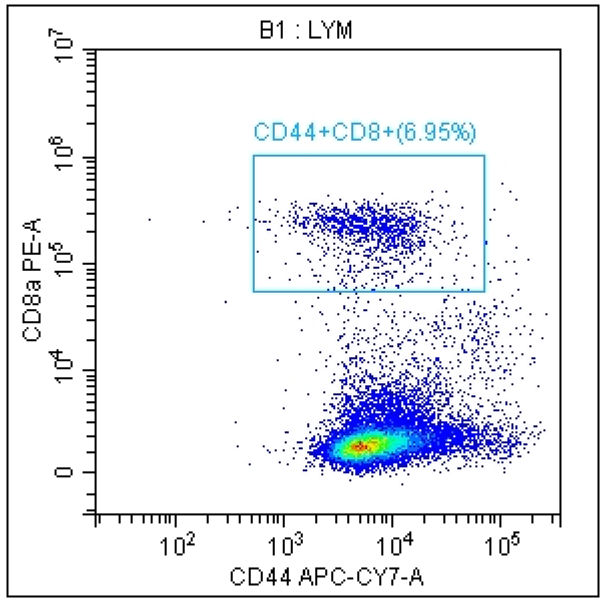

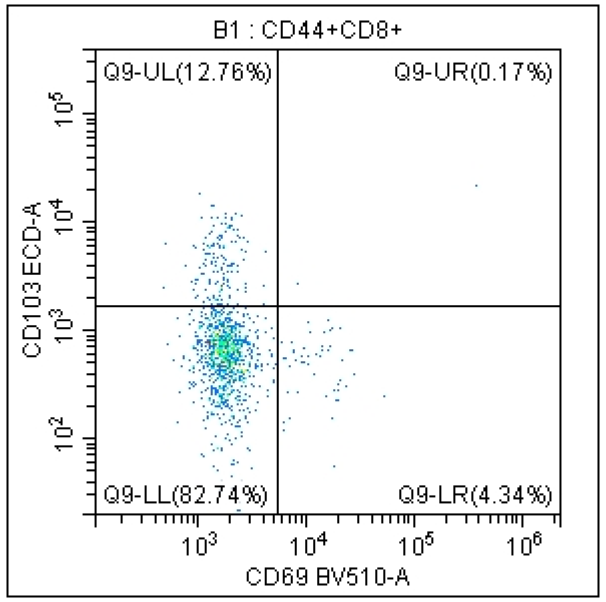

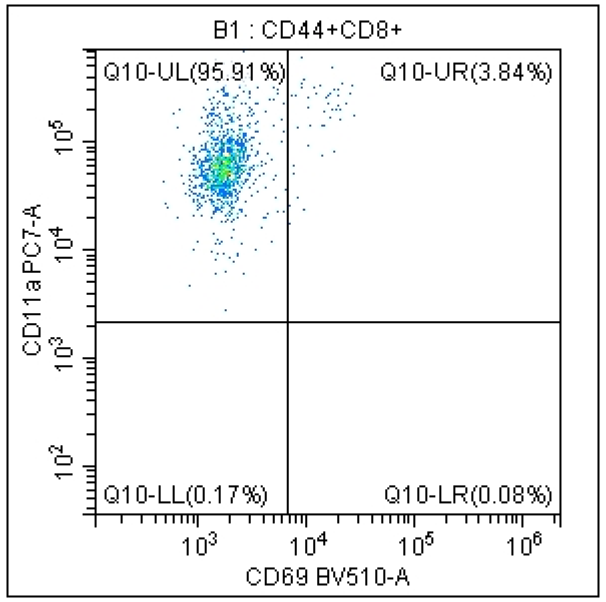

试管名称： B1

样本ID：

群体	颗粒数	%父群	%总数
All Events	48041	100.00%	100.00%
P1	34325	71.45%	71.45%
LYM	17253	50.26%	35.91%
CD3+CD4+	4631	26.84%	9.64%
CD3+CD8+	1232	7.14%	2.56%
B220+	8019	46.48%	16.69%
P2	121	2.61%	0.25%
P3	54	4.38%	0.11%
Q1-UR	250	5.40%	0.52%
Q1-UL	4379	94.56%	9.12%
Q1-LR	1	0.02%	0.00%
Q2-UR	261	21.19%	0.54%
Q2-UL	970	78.73%	2.02%
Q2-LR	1	0.08%	0.00%
Q3-UR	261	5.64%	0.54%
Q3-UL	1	0.02%	0.00%
Q3-LR	4364	94.23%	9.08%
Q4-UR	279	22.65%	0.58%
Q4-UL	14	1.14%	0.03%
Q4-LR	928	75.32%	1.93%
Q5-UR	14	0.30%	0.03%
Q5-UL	120	2.59%	0.25%
Q5-LR	196	4.23%	0.41%
Q6-UR	4	0.32%	0.01%
Q6-UL	54	4.38%	0.11%
Q6-LR	177	14.37%	0.37%
CD44+CD4+	4720	27.36%	9.82%
CD44+CD8+	1199	6.95%	2.50%
B220+CD69+	192	2.39%	0.40%
Q8-UR	178	3.77%	0.37%
Q8-UL	4539	96.17%	9.45%
Q8-LR	1	0.02%	0.00%
Q10-UR	46	3.84%	0.10%
Q10-UL	1150	95.91%	2.39%
Q10-LR	1	0.08%	0.00%
Q7-UR	48	1.02%	0.10%
Q7-UL	209	4.43%	0.44%
Q7-LL	4270	90.47%	8.89%
Q7-LR	193	4.09%	0.40%
Q9-UR	2	0.17%	0.00%

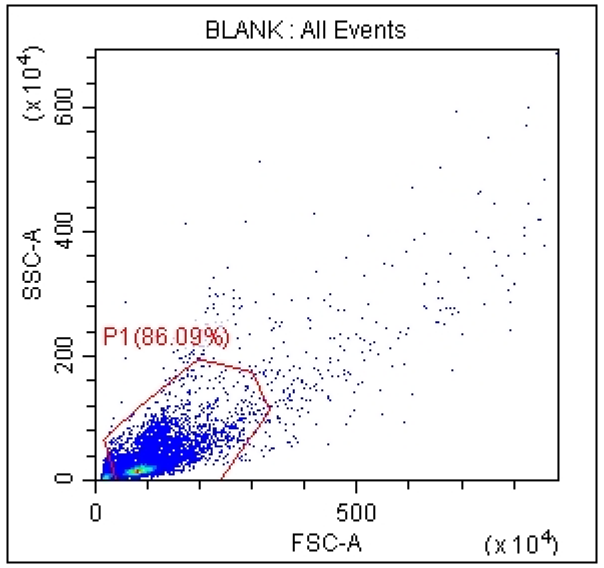

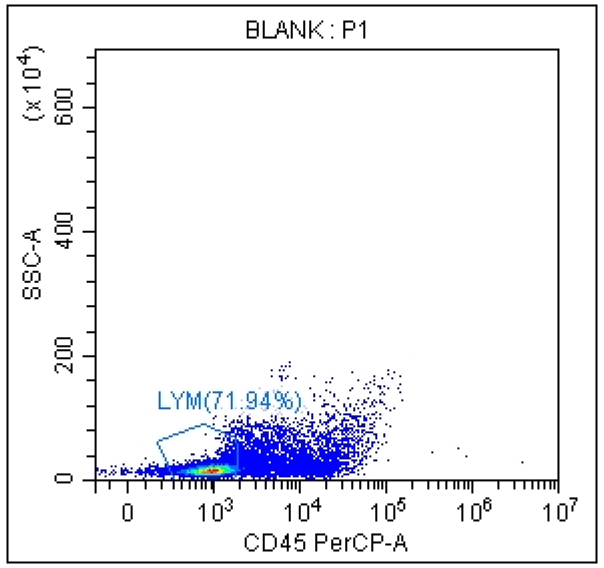

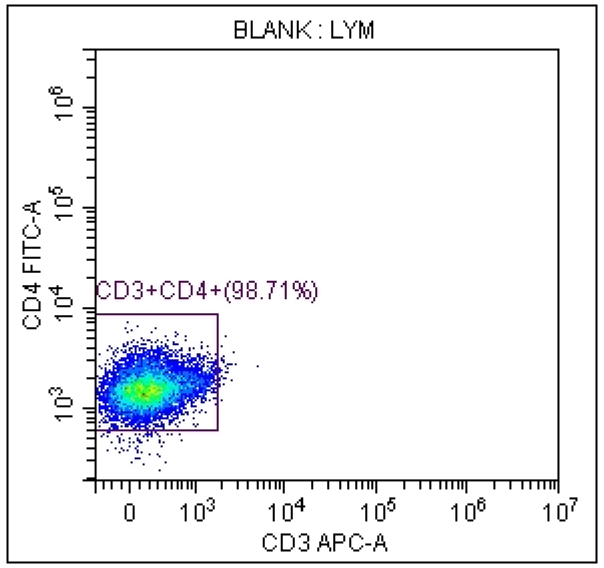

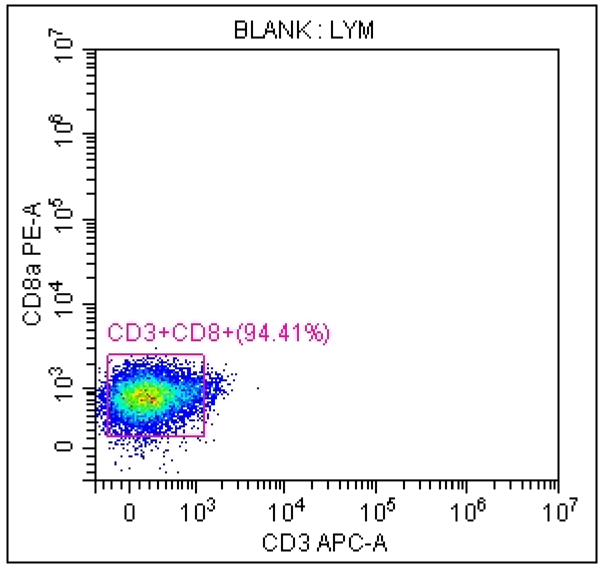

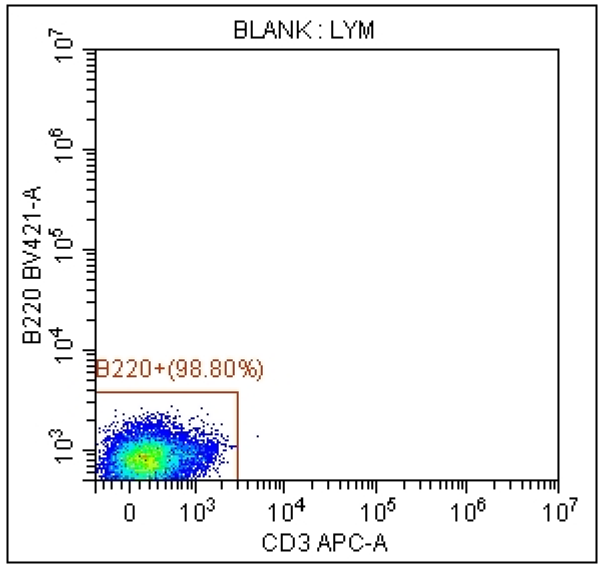

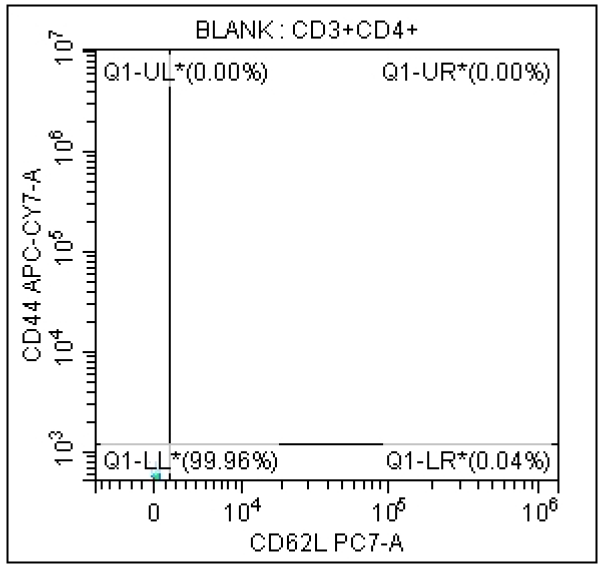

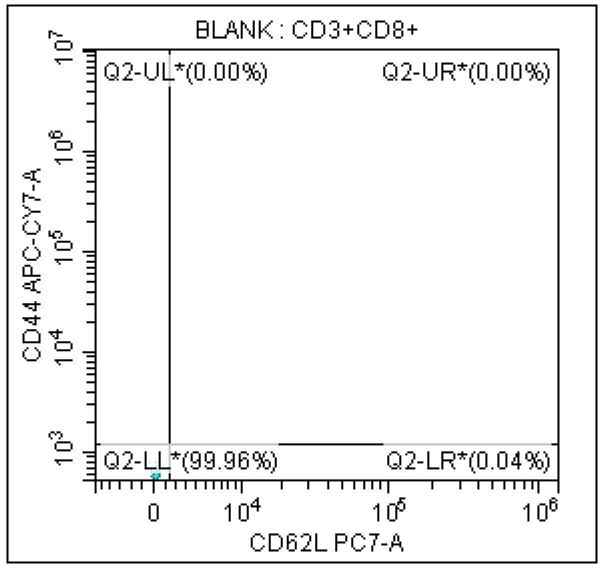

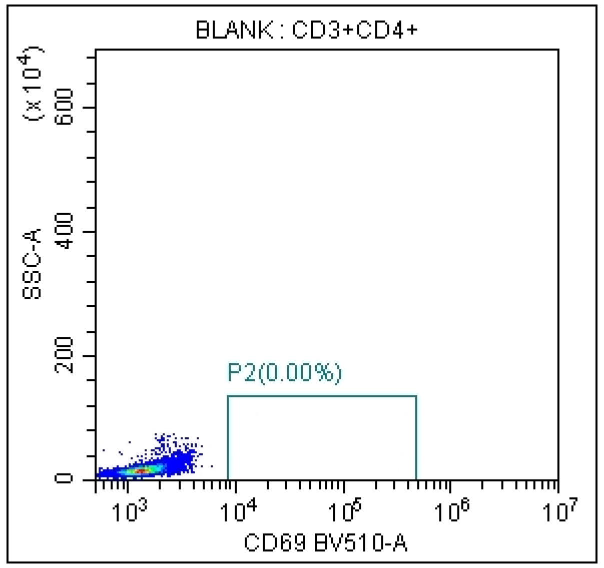

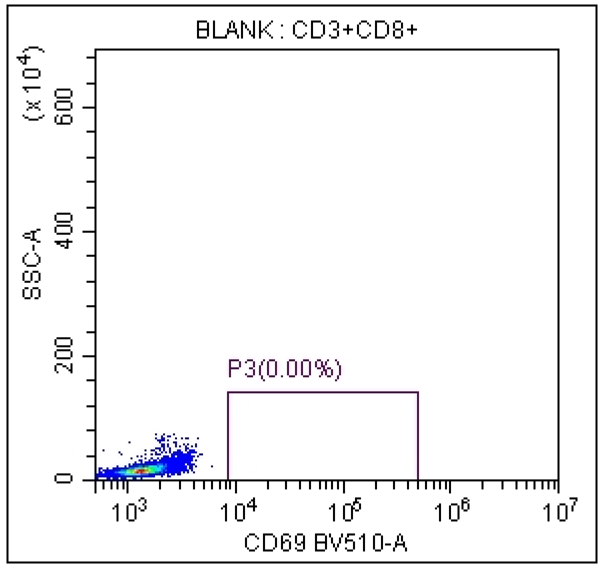

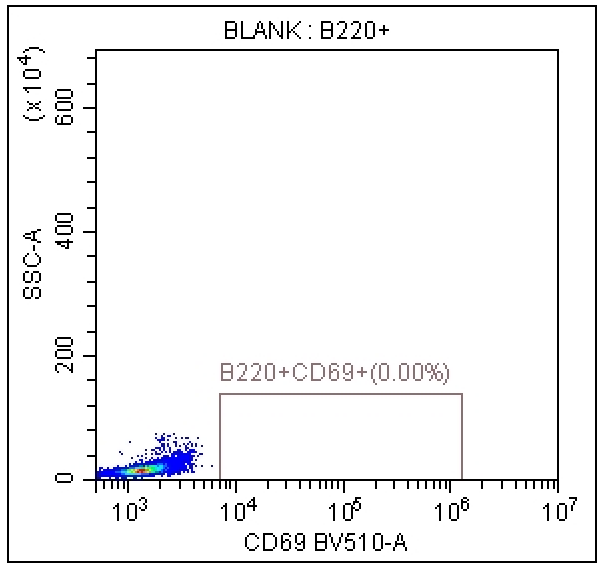

试管名称： BLANK
样本ID：

群体	颗粒数	%父群	%总数
All Events	16146	100.00%	100.00%
P1	13900	86.09%	86.09%
LYM	10000	71.94%	61.93%
CD3+CD4+	9871	98.71%	61.14%
CD3+CD8+	9441	94.41%	58.47%
B220+	9880	98.80%	61.19%
Q1-UR	0	0.00%	0.00%
Q1-UL	0	0.00%	0.00%
Q1-LL	9867	99.96%	61.11%
Q1-LR	4	0.04%	0.02%
Q2-UR	0	0.00%	0.00%
Q2-UL	0	0.00%	0.00%
Q2-LL	9437	99.96%	58.45%
Q2-LR	4	0.04%	0.02%
P2	0	0.00%	0.00%
P3	0	0.00%	0.00%
B220+CD69+	0	0.00%	0.00%
CD4+CD44+	0	0.00%	0.00%
CD8+CD44+	0	0.00%	0.00%

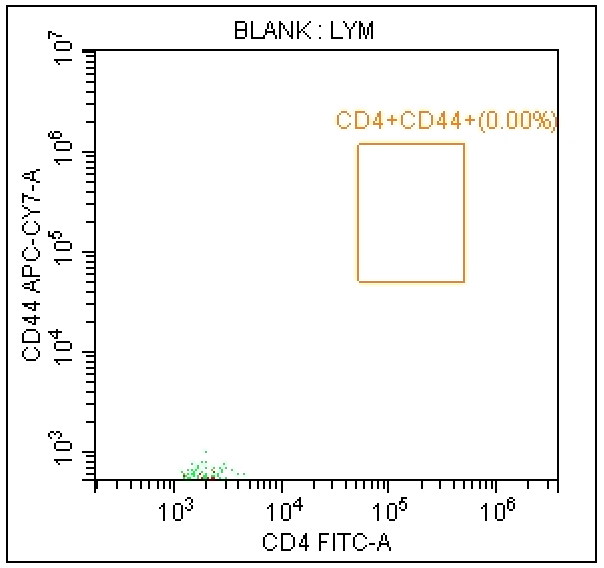

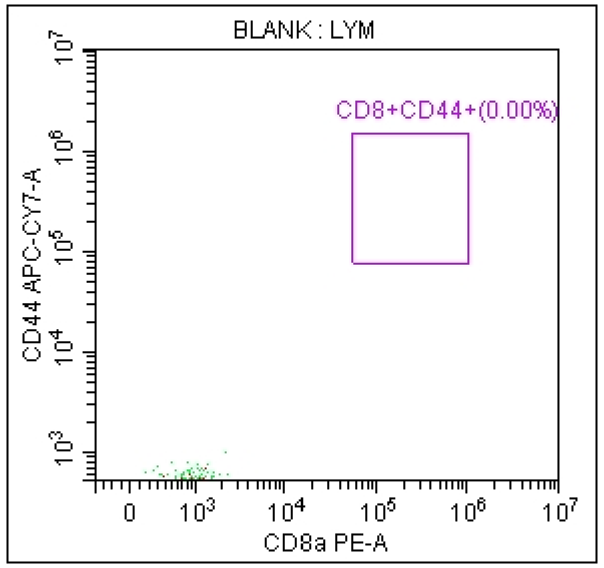

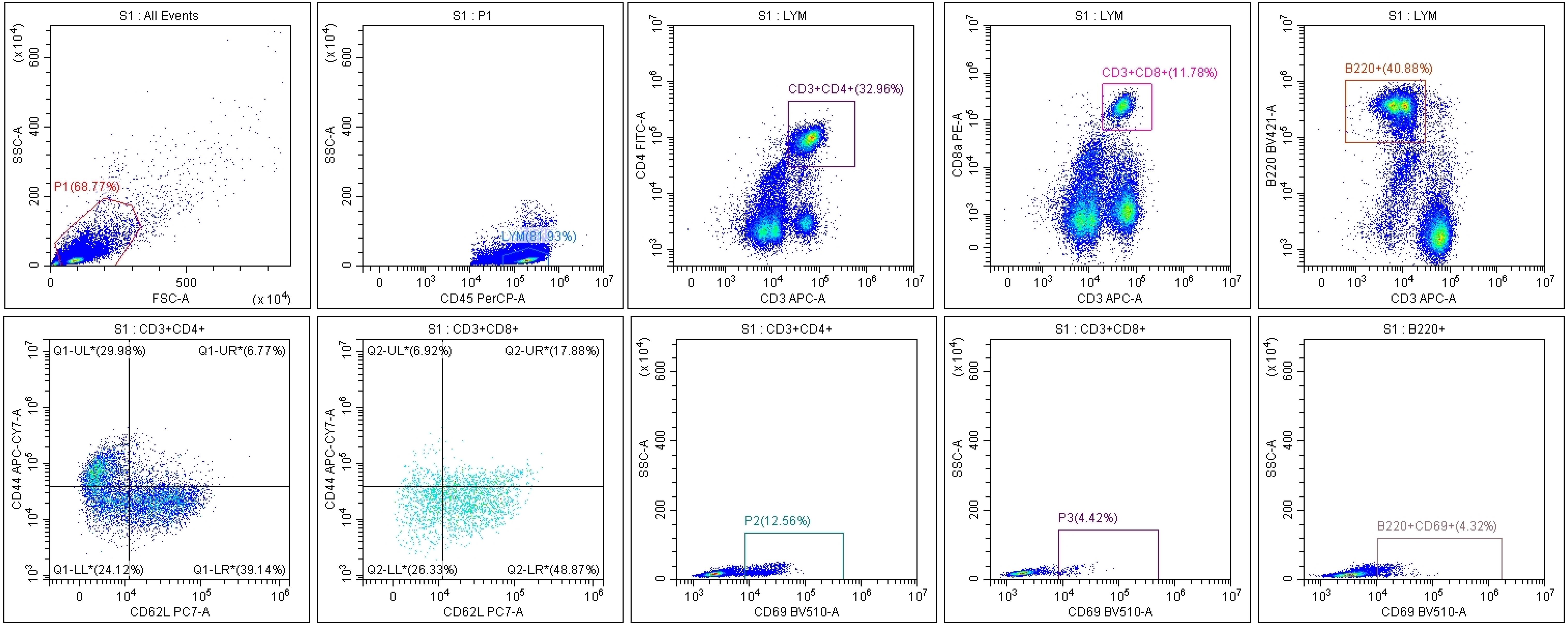

试管名称： S1

样本ID：

群体	颗粒数	%父群	%总数
All Events	35496	100.00%	100.00%
P1	24412	68.77%	68.77%
LYM	20000	81.93%	56.34%
CD3+CD4+	6592	32.96%	18.57%
CD3+CD8+	2355	11.77%	6.63%
B220+	8175	40.87%	23.03%
Q1-UR	446	6.77%	1.26%
Q1-UL	1976	29.98%	5.57%
Q1-LL	1590	24.12%	4.48%
Q1-LR	2580	39.14%	7.27%
Q2-UR	421	17.88%	1.19%
Q2-UL	163	6.92%	0.46%
Q2-LL	620	26.33%	1.75%
Q2-LR	1151	48.87%	3.24%
P2	828	12.56%	2.33%
P3	104	4.42%	0.29%
B220+CD69+	353	4.32%	0.99%

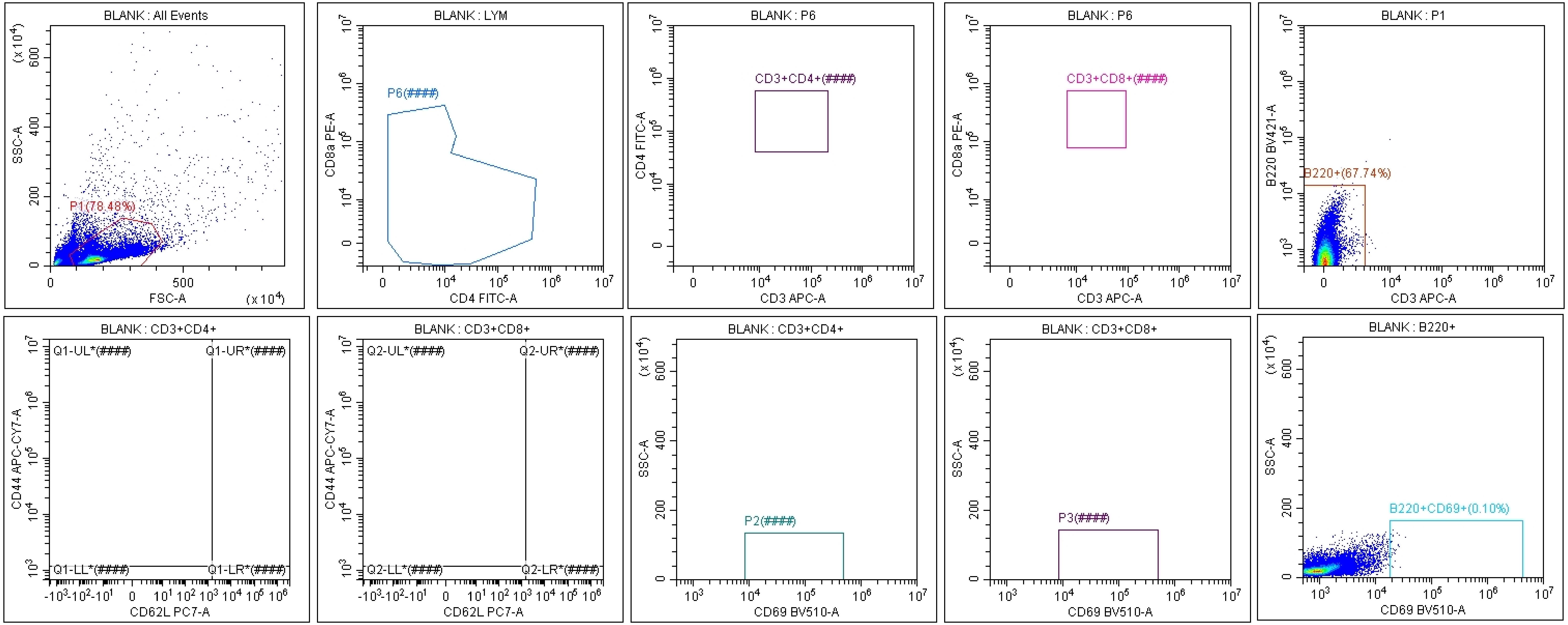

试管名称： BLANK

样本ID：

群体	颗粒数	%父群	%总数
All Events	56679	100.00%	100.00%
P1	44483	78.48%	78.48%
LYM	0	0.00%	0.00%
CD3+CD4+	0	####	0.00%
CD3+CD8+	0	####	0.00%
B220+	30132	67.74%	53.16%
Q1-UR	0	####	0.00%
Q1-UL	0	####	0.00%
Q1-LR	0	####	0.00%
Q2-UR	0	####	0.00%
Q2-UL	0	####	0.00%
Q2-LR	0	####	0.00%
P2	0	####	0.00%
P3	0	####	0.00%
CD3+	0	####	0.00%
P6	0	####	0.00%
B220+CD69+	30	0.10%	0.05%

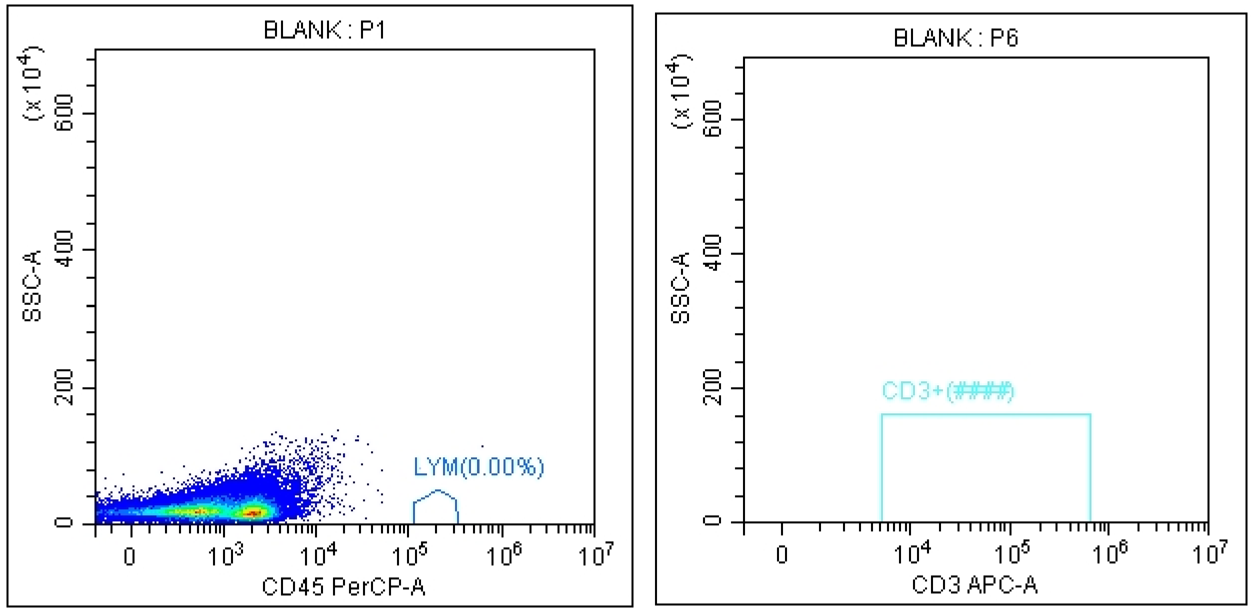

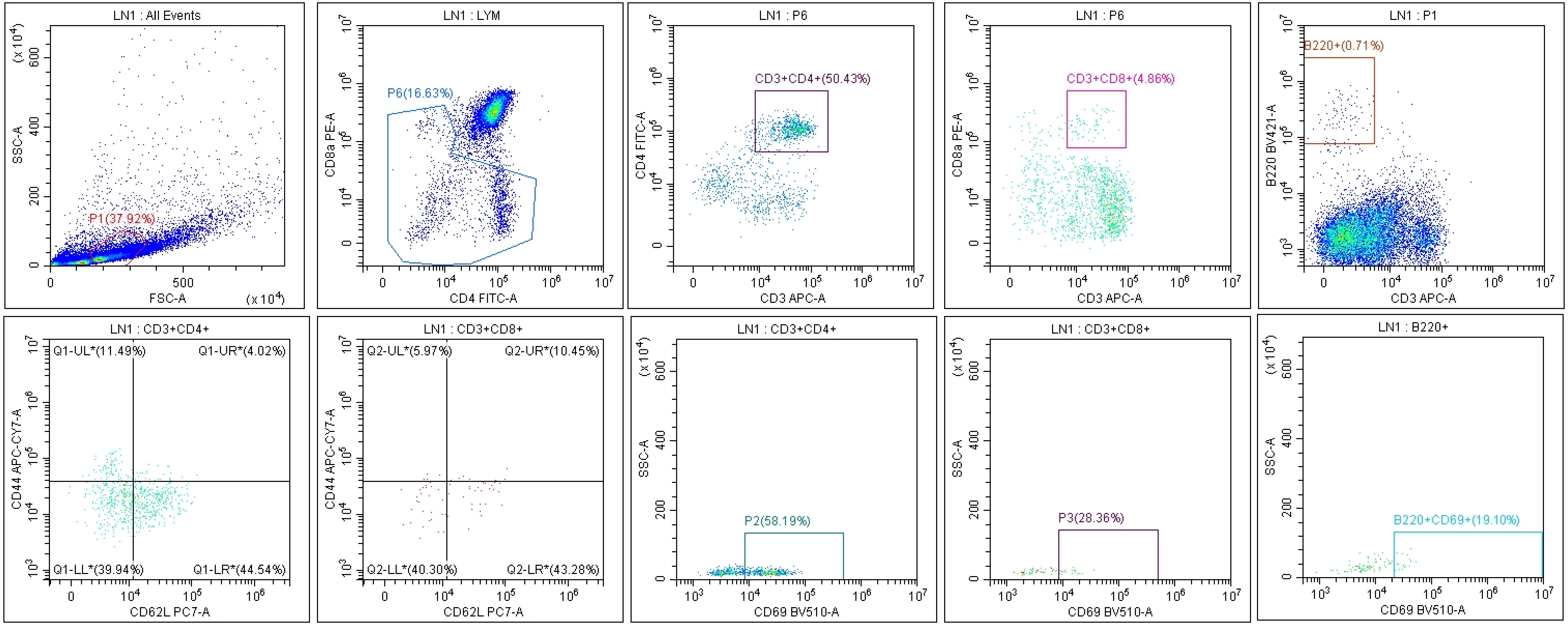

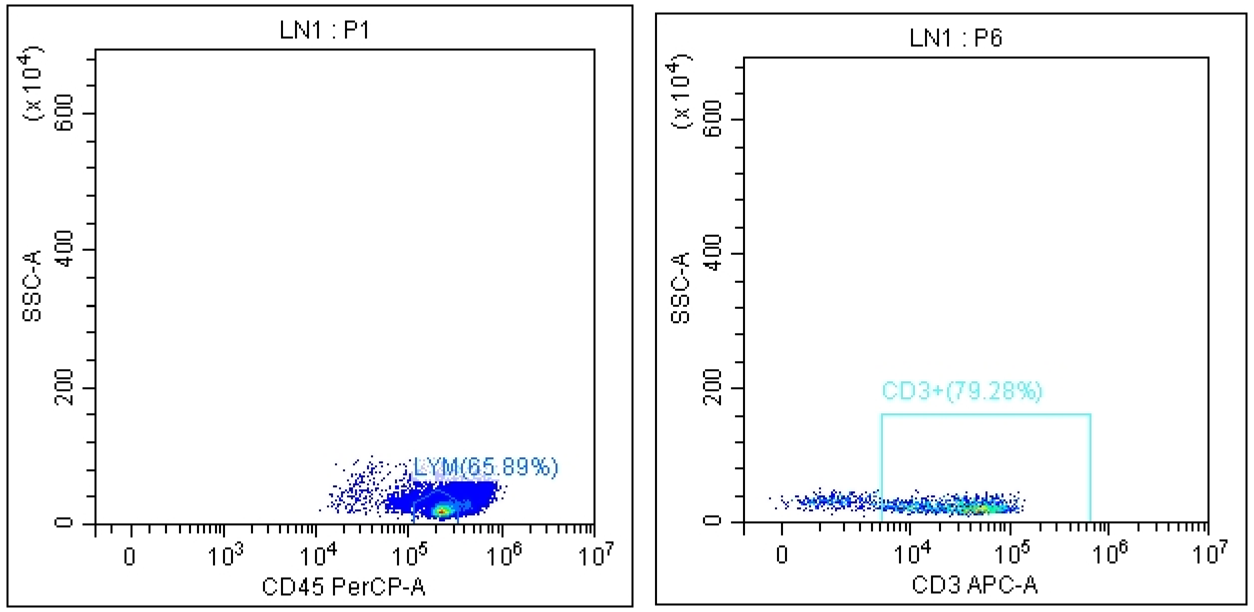

试管名称： LN1

样本ID：

群体	颗粒数	%父群	%总数
All Events	33207	100.00%	100.00%
P1	12593	37.92%	37.92%
LYM	8298	65.89%	24.99%
CD3+CD4+	696	50.43%	2.10%
CD3+CD8+	67	4.86%	0.20%
B220+	89	0.71%	0.27%
Q1-UR	28	4.02%	0.08%
Q1-UL	80	11.49%	0.24%
Q1-LR	310	44.54%	0.93%
Q2-UR	7	10.45%	0.02%
Q2-UL	4	5.97%	0.01%
Q2-LR	29	43.28%	0.09%
P2	405	58.19%	1.22%
P3	19	28.36%	0.06%
CD3+	1094	79.28%	3.29%
P6	1380	16.63%	4.16%
B220+CD69+	17	19.10%	0.05%
